# Design, synthesis, and evaluation of a pyrazole-based corrosion inhibitor: a computational and experimental study

**DOI:** 10.1038/s41598-024-76300-5

**Published:** 2024-10-24

**Authors:** Abdelmalek Matine, Bouchra Es-Sounni, Mohamed Bakhouch, Ali H. Bahkali, Habib El Alaoui El Abdallaoui, Shifa Wang, Asad Syed, Ling Shing Wong, Na’il Saleh, Abdellah Zeroual

**Affiliations:** 1 Molecular Modeling and Spectroscopy Research Team, Faculty of Sciences, Chouaib Doukkali University, PB 20, 24000 El Jadida, Morocco; 2 Bioorganic Chemistry Team, Department of Chemistry, Faculty of Sciences, Chouaïb Doukkali University, P.O. Box 24, 24000 El Jadida, Morocco; 3 Department of Botany and Microbiology, College of Science, King Saud University, Riyadh, Saudi Arabia; 4 School of Electronic and Information Engineering, Chongqing Three Gorges University, Chongqing, Wanzhou, 404000 China; 5 Faculty of Health and Life Sciences, INTI International University, Putra Nilai, 71800 Nilai, Negeri Sembilan Malaysia; 6 Department of Chemistry, College of Science, United Arab Emirates University, P.O. Box 15551, Al Ain, United Arab Emirates

**Keywords:** Pyrazole derivative, Corrosion, Electrochemical assessments, DFT, Langmuir adsorption isotherm, Radial distribution function, Electrochemistry, Corrosion, Molecular dynamics

## Abstract

By employing a synergistic blend of experimental and theoretical methodologies, we investigated the corrosion inhibition efficacy of a synthesized pyrazole derivative (BM-01) in a solution of hydrochloric acid (1 M). We utilized molecular dynamics (MD) simulations, scanning electron microscopy (SEM), density functional theory (DFT), complexation, plus electrochemical impedance spectroscopy (EIS). We conducted weight loss (WL) measurements from 298 to 328 K. Inhibition efficacy reached a maximum at a BM-01 concentration of 10^−3^ M, achieving 90.0% (EIS), 90.40% (WL), and 90.38% (potentiodynamic polarization (PDP)). SEM unveiled the shielding of the carbon-steel surface from acid-induced damage by BM-01. The Langmuir adsorption isotherm exhibited a robust fit with a low sum of squares, standard deviation, and a high correlation coefficient. PDP findings indicated that BM-01 acted as a mixed-type inhibitor, predominantly favoring the cathodic process, suggesting potential corrosion-mitigation properties. Theoretical analyses involving DFT, MD simulations, and radial distribution function were conducted to postulate a mechanism and identify an inhibitory layer. Theoretical outcomes aligned closely with experimental data, thereby reinforcing the validity of our findings.

## Introduction

Industries face a significant challenge in the form of material corrosion. Material corrosion presents significant risks to the functionality and longevity of metallic equipment, thereby reducing productivity and diminishing profitability in these sectors^1^. Given their affordability and excellent mechanical properties, metallic materials are employed widely in industrial and technological fields. Carbon steel, in particular, is employed in: marine applications; chemical industries; construction of facilities for production and refinement of oil; manufacture of metal equipment. Carbon steel is also used for the long-distance transportation of acid solutions and petrochemicals, but the corrosive nature of these substances causes damage to the inner surfaces of pipelines during transportation^2^.

Acid corrosion poses a significant challenge in chemical manufacturing, maritime, and petrochemical industries. Acidic solutions find extensive application across various industrial processes, including chemical cleaning and treatment, steel pickling, and mineral extraction^3^. Due to their aggressive nature, these acidic solutions contribute to corrosion on the inner surfaces of metallic components during these processes, thereby presenting risks to public safety, environmental concerns, and substantial financial losses. Consequently, mitigating and controlling metal corrosion is very important^4^.

In recent years, pyrazole-based inhibitors have attracted significant attention for their therapeutic potential and applications in adsorption^5^. These compounds effectively target enzymes and receptors associated with diseases, exhibiting anti-inflammatory, analgesic, anticancer, and antimicrobial properties. Additionally, pyrazole derivatives are being investigated for treating diabetes, neurodegenerative disorders, cardiovascular diseases, and for adsorption applications due to their interaction capabilities^6^. Ongoing research aims to optimize their chemical structures to enhance efficacy and selectivity.

Pyrazole-based inhibitors are employed widely to mitigate corrosion. They form a protective physical barrier layer on the metal substrate through their adsorption^5^. Typically added in small quantities to minimize environmental reactivity, the efficiency and adsorption ability of inhibitors are tied intricately to their molecular structure, spatial geometry, the nature of their functional groups, and attractive forces. Various chemicals (whether produced synthetically or occurring naturally in organic compounds) are selected for their polar characteristics, which encompass the existence of heteroatoms (e.g., nitrogen (N), oxygen (O)) and/or π-systems^6^. The involvement of heteroelements is considered crucial in the adsorption of these compounds onto metal surfaces^7^. Consequently, the behavior of an inhibitor candidate to stop corrosion determines its choice, and often is linked to its structure. Thus, an excellent corrosion inhibitor should contain multiple active absorption centers, such as heteroatoms (e.g., N, O, sulfur (S)) and aromatic rings.

Pyrazole heterocycles find applications as herbicides, fungicides, insecticides, and dyes. Pyrazole heterocycles have been shown to have anti-inflammatory, antidepressant, antibacterial, and antispasmodic properties^8^. Such findings align with our ongoing research, given the pivotal role these chemicals have in biological functions. Our research team aim to discover effective inhibitors to mitigate metal corrosion in challenging conditions, particularly those involving hydrochloric acid (HCl).

Organic compounds have been used extensively for the prevention of and resistance to corrosion^9^. Among organic compounds, heterocycles with motifs such as isoxazole^10^, isoxazoline^11^, pyrazole^11^, and pyrazoline^12^ have received considerable attention.

We synthesized and characterized 1,3,5-triphenyl-4,5-dihydro-1H-pyrazole (BM-01) and evaluated its capabilities to inhibit corrosion. Potentiodynamic polarization (PDP) tests in a HCl solution (1 M) and electrochemical impedance spectroscopy (EIS) were undertaken. Subsequent to corrosion studies, the steel surface underwent examination using scanning electron microscopy (SEM).

We wished to enhance understanding of the interaction between BM-01 and corrosive substances at the metal interface. Hence, molecular dynamics (MD) modeling and density functional theory (DFT) calculations were utilized for comprehensive computational assessments to complement our experimental investigations. In particular, DFT simulations were employed to gain a detailed insight into the local and global reactivity of BM-01.

## Experimental section

### Synthesis of BM-01

Each reagent matched the requirements for analytical purity and was purchased from commercial vendors. A bench device (Kofler Energies, Berlin, Germany) was used to determine the melting point. A Fourier transform infrared (FTIR) spectrometer (Vertex 70; Bruker, Billerica, MA, USA) was used for FTIR spectroscopy. We employed a 300-MHz nuclear magnetic resonance (NMR) Bruker to obtain ^1^H and ^13^C NMR spectra. The solvent applied was CDCl_3_, whereas Tetramethylsilane (TMS) was chosen as the internal standard. Values for the proton coupling constant (J) are denoted in Hertz, and chemical shifts (δ) are in parts per million. Spin multiplicities were singlet, triplet, multiplet, doublet of doublets, and triplet. Chalcone 1 was produced via a conventional method^13^.

Chalcone (2 mol) was solubilized in 60 ml of acetic acid in a 200-ml condensing flagon. To this solution was added phenylhydrazine (2 mol). Thin-layer chromatography was used to monitor the reaction mixture while it was heated under reflux for several hours. After the reaction had been completed, the mixture was placed on cold water. The resulting precipitate was passed through a 0.45 micron filter and washed thoroughly. The isolated material was recrystallized in ethanol at a melting point of 140–142 °C to produce the required pyrazole, BM-01, with a yield of 78%.

Attenuated total reflection-infrared (ATR-IR) spectroscopy (neat, ω in cm^−1^) revealed peaks at 1594 (C=N), 1503, 1488, 1455, and 1445 (C=C–C=C), and 3030 (C_a_–H). ^1^H NMR (300 MHz, CDCl₃, δ in ppm) revealed peaks at 31.7 (1H, dd, J^trans^ = 8.29 Hz, H₄, J^gem^ = 17.14 Hz), 3.87 (1H, dd, H₄, J^cis^ = 12.4 Hz, J^gem^ = 18.01 Hz), 5.30 (1H, dd, H₅, J^trans^ = 7.28 Hz, J^cis^ = 12.35 Hz), and 6.78–7.99 (m, 15H, Har). ^13^C NMR (75 MHz, CDCl_3_, δ in ppm) revealed peaks at 43.59 (C4), 64.52 (C5), 105.19, 113.38, 119.10, 125.47, 125.74, 125.88, 125.99, 127.57, 128.54, 128.59, 128.70, 128.77, 128.90, 128.97, 129.14, 132.75 (C3-C_Ar_), 142.60 (C5-C_Ar_), 144.79 (N-C_Ar_), 146.71 (C3).

### “Mild” steel and solution

We utilized samples of carbon steel with specific chemical compositions: silicon (0.230%), titanium (0.011%), S (0.016%), cobalt (0.009%), manganese (0.680%), chromium (0.077%), copper (0.160%), carbon (0.370%), nickel (0.059%), and iron (Fe) as a balance. Subsequently, a HCl solution (1 M) was prepared by diluting commercial acid (37% HCl) with twice-distilled water. Concentrations ranging between 10^−3^ and 0.5 10^−4^ M were employed in electrochemical experiments and weight-loss studies, respectively.

### Weight loss

Reduction in the weight of specimens of carbon steel was assessed by comparing their weights before and after immersion in HCl solution (1 M) with various concentrations of BM-01 at 298 K for 13 h employing a high-precision balance. The corrosion rate (W) was calculated using Eq. (1), followed by the determination of the inhibition efficacy (E%) of BM-01 through Eq. (2)^14^.

1 $${\text{W}} = \frac{{{\text{m}}_{{\text{i}}} - {\text{m}}_{{\text{f}}} }}{{{\text{s}}*\Delta {\text{t}}}}$$

2 $${\text{E\% }} = \frac{{{\text{W}}_{{{\text{blan}}}} - {\text{W}}_{{{\text{in}}}} }}{{{\text{W}}_{{{\text{blan}}}} }}*100$$

The rate of iron corrosion in a HCl solution (1 M) at various BM-01 concentrations with and without an inhibitor was represented by the variables W_in_ and W_blan_, respectively. They were represented by the initial and final weights, the surface area of carbon steel, and the immersion period as, mi, mf, S, and t, respectively.

### Measurement of the electrochemical reaction

An experimental arrangement utilized a potentiostat/galvanostat (VersaSTAT3; Ametek, Berwyn, PA, USA) controlled by a computer running VersaStudio software to conduct polarization curves and measurements of electrochemical impedance. Current–potential curves were obtained using potentiodynamic mode at a scan speed of 1 mV/s. The sample potential showed a constant fluctuation in the range − 800 to − 200 mV/ECS^15^.

This device for electrochemical measurement comprised three electrodes: platinum counter, carbon steel, and Ag/AgCl reference. After the open circuit potential (OCP) had reached a stable state, electrochemistry tests were carried out. EIS was employed in a frequency range between 100 kHz to 10 MHz during the OCP^16^. Equation 3 was used to determine the effectiveness of the inhibitor according to the resistance to charge transfer^17^.

3 $$\eta \% = \frac{{R_{ct}^{i} - R_{ct}^{^\circ } }}{{R_{ct}^{i} }} *100$$

where the resistance to charge transfer is denoted by R^°^_ct_ and R^i^_ct_ in the presence or absence of an inhibitor, respectively.

PDP measurements were done under inhibition and non-inhibition conditions on the surface of mild steel. Before the sweeps, an alternating current (AC) impedance test was conducted at a rate of 0.5 mV/s. Potentiodynamic data were analyzed using a polarization device (VoltaMaster; Informer Technologies, Los Angeles, CA, USA). The corrosion potential (E_corr_) and corrosion current density (I_corr_) were obtained by extrapolating the linear segments of Tafel plots from anodic and cathodic curves. Subsequently, curve fitting was applied to determine the I_corr_ based on the polarization curves generated by Eq. (4)^18^:

4 $${\text{I}} = {\text{I}}_{{{\text{corr}}}} \left[ {\exp \left( {2.3\Delta {\text{E/}}\beta_{{\text{a}}} } \right) - \exp \left( {2.3\Delta {\text{E/}}\beta_{{\text{c}}} } \right)} \right]$$

This correlation was employed to compute the inhibition efficacy based on the obtained I_corr_ values^19^:

5 $$\eta \left( \% \right) = \frac{Icorr - Icorr\left( i \right)}{{Icorr}}*100$$

where the corrosion current density aimed at the carbon-steel electrode in a solution with and without inhibition is represented by I_corr(i)_ and I_corr_, respectively.

### Surface examination: SEM

The surface properties of C38 steel plates were investigated using a scanning electron microscope (S-570; Hitachi, Tokyo, Japan). Surface features were identified using a flash X-ray analyzer (6130; Bruker) operating at an accelerating voltage of 20 kV. C38 steel plates were immersed for 12 h at 298 K in a solution of HCl (1 M) with or without BM-01 (10^−3^ M), then quenched for an additional 12 h. Finally, they were dried and cleaned.

### Conceptual details

Calculations involving quantum chemistry have been employed in corrosion studies to ascertain the electronic structure of organic molecules. This approach enables assessment of the inhibition efficacy of an organic molecule by elucidating its adsorption mechanism on the steel surface. Essential descriptors encompass highest occupied molecular orbital (HOMO), lowest occupied molecular orbital (LUMO), gap energy, dipole moment, and ∆N110 energy. These values were determined using Gaussian 09 W software^20,21^.

BM-01 was optimized utilizing DFT/B3LYP, which provides exceptional precision and accuracy in representing organic molecules. The optimization employs the 6-311G(d, p) basis set^22^.

### MD and Monte Carlo simulations

An effective method for examining intramolecular interactions is MD simulation (especially if metals and inhibitors are involved)^23^. After evaluating various designs, we selected the Fe (110) design as the most suitable for our investigation. To enhance the surface area for studying inhibitor action, the Fe (110) surface was expanded using a supercell of (10). A zero-thickness vacuum layer was generated^23^. The MD simulation employed optimized operating settings, including an NVT ensemble (in which simulated particles (N), simulation cell volume (V) and temperature (T) are all kept fixed throughout the simulation) with an Andersen thermostat, simulated box containing periodic boundaries of 30.18 × 42.42 × 45.57^24^, and a vacuum layer (20) with 1 BM-01, 15 H_3_O^+^, 15 Cl^−^, and 233 H_2_O within it.

The following parameters were used in the experiment: temperature maintenance at 203 K and 343 K; application of the COMPASS force field; implementation of a phase duration of 1 fs; extension of the simulation time to 500 ps. Equations (6) and (7) were utilized to determine the binding energy (E_Binding_) and interaction energy (E_Interaction_) of the system^25^.

6 $$E_{{\text{int}}} = E_{complix} - \left( {E_{inh} + E_{{Fe + 233H_{2} O + 15Cl^{ - } + 15H_{3} O^{ + } }} } \right)$$

7 $$E_{bind} = - E_{{\text{int}}}$$

where E_Fe + H2O + H3O_^+^_+ Cl_^−^ denotes the energy of the ferric interface in the presence of H_2_O, H_3_O^+^ and Cl^−^, E_complix_ denotes the total energy of the simulated structure, and E_int_ represents the free energy of the inhibitor molecule.

## Results and discussion

### Synthesis

We employed a method documented in the literature^26^ to synthesize the pyrazole BM-01. Phenylhydrazine was condensed with chalcone 1 in acetic acid upon reflux for 1 h. Addition of a primary amine was triggered by a Michael 1,2-addition reaction on the carbonyl group to form the Michael adduct. BM-01 was obtained by combining the α-carbon of chalcone 1 with a secondary amine (NH) group (Fig. S1). Chalcone 1 was prepared according to a method mentioned previously^13^. The structural characteristics of BM-01 were ascertained through NMR and IR spectroscopy. ATR-IR spectrum (Fig. S2) revealed a distinct absorption band at 1593 cm^−1^, which signified a N=C bond within the pyrazole ring. Vibrating of the aromatic C–H bonds of the aromatic ring was observed at 3035 cm^−1^. Notably, two bands pairs between 1450 and 1508 cm^−1^ corresponded to vibrations within the aromatic backbone.

**Fig. 1:**
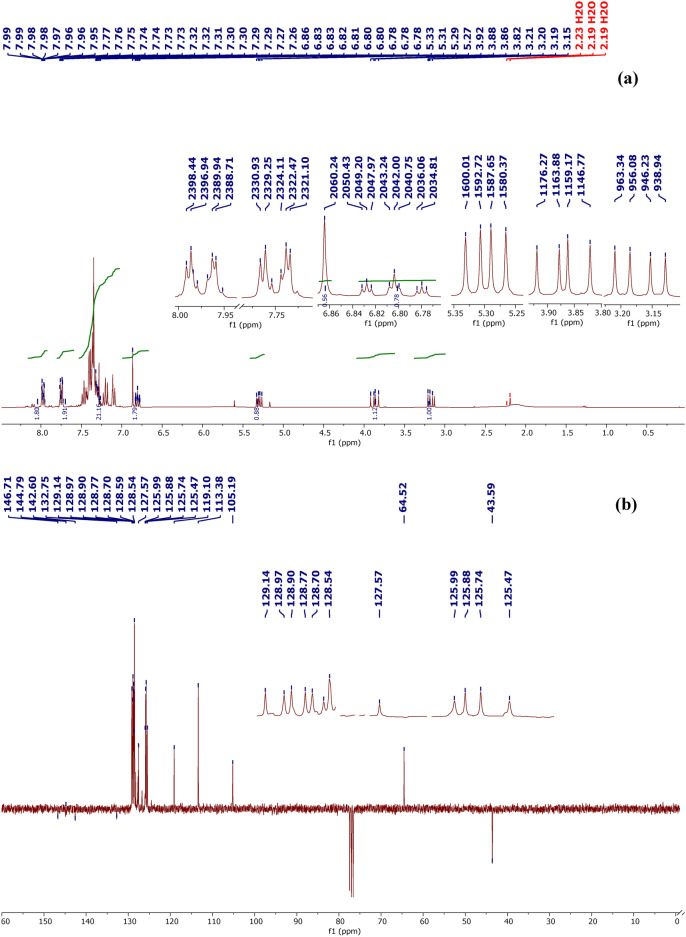
(**a**) ^1^H NMR (300 MHz) and (**b**) ^13^C NMR (APT, 75 MHz) spectra of pyrazole BM-01.

In addition to the aromatic proton signals between 6.78 and 7.99 ppm, the ^1^H NMR spectrum of BM-01 in CDCl_3_ (Fig. 1) exhibited three characteristic doublets of a doublet of an ABX spin system positioned at 3.17, 3.87, and 5.29 ppm, with coupling constants 3Jax = 7.3 Hz, 3Jbx = 12.4 Hz and Jab = 17,1, respectively. These doublets of a doublet corresponded to the three protons attached to the C4 and C5 carbon atoms of the pyrazole ring. In addition, the ^13^C NMR spectrum of BM-01 (Fig. 1) revealed a signal at 43.58 ppm corresponding to carbon C4, 64.52 ppm (assigned to carbon C5), and 158.62 ppm (associated with carbon C3). Moreover, signals observed in the spectrum at 132.75, 142.60 and 144.49 ppm, corresponded to carbon atoms at positions 3, 5, and 1 of phenyl rings.

### Gravimetry

#### Concentration effect

Understanding inhibitor–metal interactions starts with calculating the average corrosion rate directly using a gravimetric approach.

Carbon steel was immersed in a corrosive solution for 12 h without or with an inhibitor at varying concentrations. The corrosion rate (W_corr_) was calculated using gravimetric methods. Equations (8 and 9) were employed to obtain the corrosion rate and percentage inhibition, respectively:

8 $$W_{corr} = \frac{\Delta m}{{t*S}}$$

9 $${\text{E\% }} = \left( {\frac{{{\text{W}}_{{{\text{blan}}}} - {\text{W}}_{{{\text{ih}}}} }}{{{\text{W}}_{{{\text{blan}}}} }}} \right)$$

Table 1 describes the W_corr_ of carbon steel and efficacy of corrosion inhibition in a HCl solution (1 M) at different temperatures and concentrations with and without an inhibitor. A higher inhibitor concentration corresponded to a lower W_corr_ and higher percent inhibition (E%). At a concentration of 10^−3^ M, the percent inhibition reached 90.4%. This improvement in inhibition efficacy could be explained by the significant adsorption of the compound to the surface of mild steel^27^. Nevertheless, the inhibition efficacy decreased with increasing temperature, probably due to the destabilized interaction between the compound and metal surface.

**Table 1:** Gravimetric properties of C38 steel immersed in HCl (1 M) under different temperatures and concentrations with and without BM-01.

Inhibitor	C (M)	298 K		308 K		318 K		328 K	
		W (mg/cm^2^ h)	E (%)	W (mg/cm^2^ h)	E (%)	W (mg/cm^2^ h)	E (%)	W (mg/cm^2^ h)	E (%)
	Black	2.12		4.23		8.32		10.65	
BM-01	5.00 × 10^–5^	0.650	69.33	1.358	67.89	2.892	65.24	3.869	63.67
	1.00 × 10^–4^	0.389	81.65	0.837	80.21	1.783	78.56	2.557	75.99
	5.00 × 10^–4^	0.381	82	0.822	80.56	1.837	77.91	2.519	76.34
	1.00 × 10^–3^	0.203	90.4	0.466	88.96	1.222	85.31	2.051	80.74

Using the results in Table 1, we plotted the variation in corrosion rate with inhibition efficacy (Fig. 1). The mass loss and corrosion rate decreased with increasing inhibitor concentration in an aggressive solution, whereas the inhibition efficacy increased with increasing recovery rate. An examination of the data presented in Table 1 and Fig. 1 revealed a consistent pattern: as the inhibitor concentration increased, the degree of corrosion (W_corr_) decreased, and the inhibition efficacy (E%) increased. The efficacy of BM-01 may have been influenced by heteroatoms and π electrons within aromatic rings.

#### Influence of temperature

**Fig. 2:**
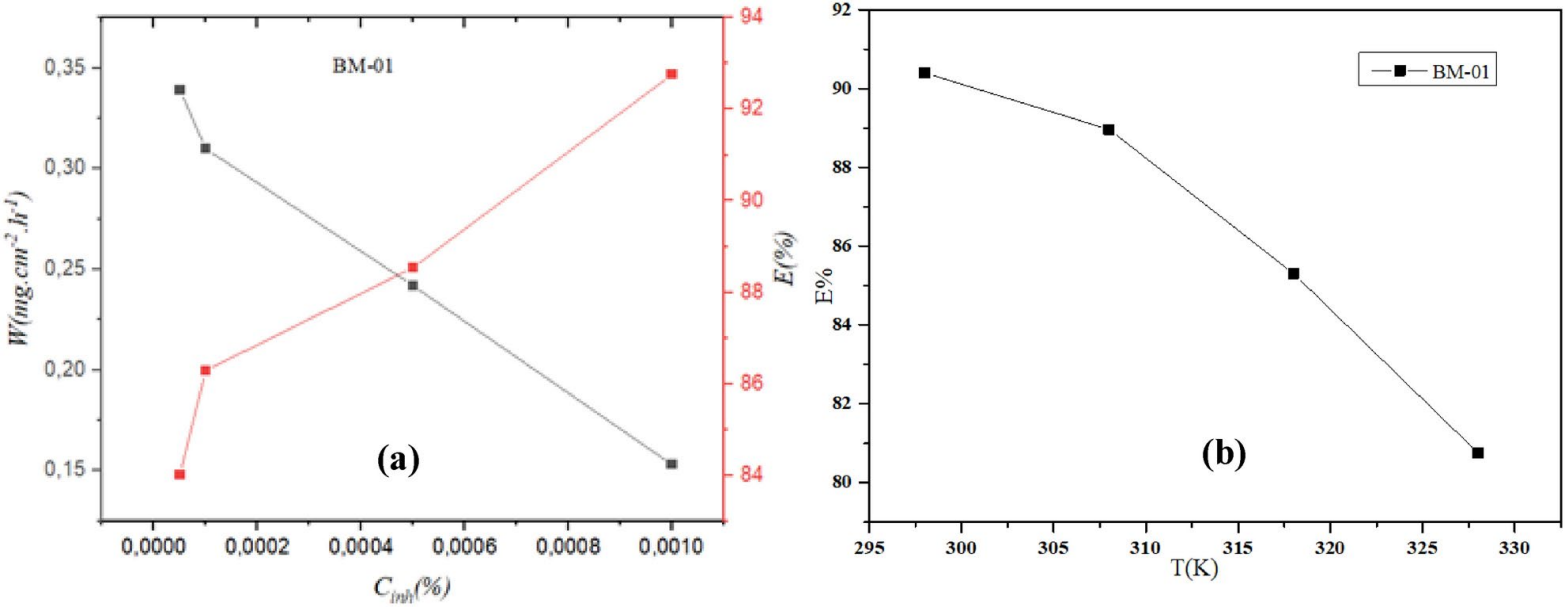
(**a**) Correlation between corrosion rate, inhibition efficiency and BM-01 concentration at room temperature 298 k. (**b**) Relation between inhibition efficiency and temperature.

Temperature plays a crucial part in altering the behavior of a material in a corrosive environment. Temperature can affect the interaction between a metal and an inhibitor in a given environment. As depicted in Fig. 2, the corrosion resistance at a concentration of 10^−3^ M decreased with temperature fluctuations from 25 to 55 °C^28^.

### Electrochemistry

#### OCP analysis

**Fig. 3:**
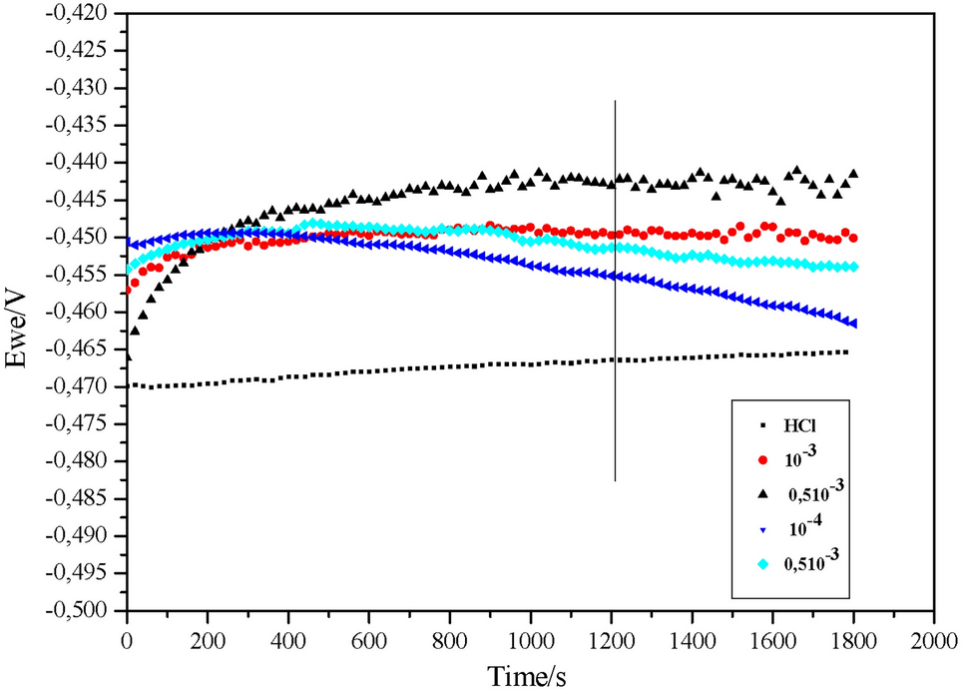
Evolution of the open circuit potential (OCP) as a function of time for carbon steel (CS) in a 1.0 M HCl solution at a temperature of 298 K.

The OCPs of carbon steel plunged into a HCl solution (1 M) for 30 min with and without BM-01 at varying concentrations were surveyed (Fig. 3). Initially, the OCP *versus* time curves were non-linear, but they eventually converged, indicating the stabilization of OCPs in each scenario^29^. Moreover, this parallel stability over time suggested the removal of the accumulated oxide layer (Fe_2_O_3_, Fe_3_O_4_) from the surface. The effect of BM-01 occurred within the first 30 min of immersion (Fig. 3).

#### Polarization

**Fig. 4:**
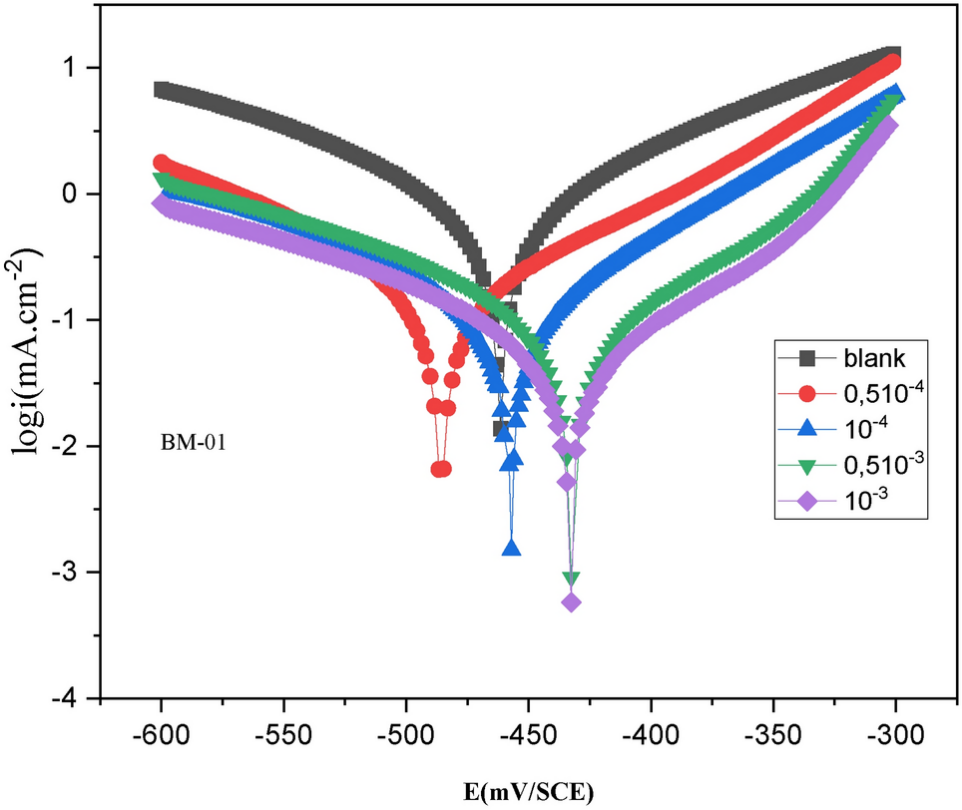
PDP curve for C38 steel in 1.0 M HCl uninhibited and inhibited by different concentrations of BM-01 at 298 K.

Polarization curves provide insights into the kinetics of anodic and cathodic reactions. They are useful tools for investigating corrosion mechanisms. Table 2 displays the electrochemical characteristics derived from the PDP curves depicted in Fig. 4. These variables included variations in cathodic (βc) and anodic (βa) Tafel slopes, E_corr_, and I_corr_. Figure 4 and Table 2 demonstrate that the cathodic polarization curves remained largely unchanged, with minor modifications observed in anodic branches. This observation extended to E_corr_ values, which showed a minor positive shift. A comparison of PDP curves revealed that inclusion of inhibitor concentrations led to a noticeable decrease in I_corr_ irrespective of whether the inhibitor was present or not. These outcomes implied that the anodic effect of dissolving metal was modified slightly by the mixed-type inhibitory activity of our pyrazole derivative. This phenomenon suggested that the inhibitor controlled the hydrogen-evolution reaction when it was active. By adsorbing at the interface between the metal and solution, the inhibitor inhibited active corrosion sites within the protective layer^30^.

**Table 2:** Various PDP parameters at 298 K for carbon steel without and with different BM-01 concentrations.

	$C \left( M \right)$	$- E_{corr} \left( {\text{mV/SCE}} \right)$	$i_{corr} \left( {\mu {\text{A/cm}}^{2} } \right)$	$\beta_{a} \left( {\text{mV/dec}} \right)$	$- \beta_{c} ({\text{mV/dec}})$	$\eta \%_{{{\text{pdp}}}}$
	Black	461.9	275.4	187.2	166.4	–
BM-01	0.5 × 10^–4^	485.19	82.62	14.95	17.15	70%
	1.0 × 10^–4^	457.66	51.499	26.54	26.36	81.3%
	0.5 × 10^–3^	432.60	49.572	15.78	19.87	82%
	1.0 × 10^–3^	433.00	27.54	24.87	21.14	90%

Table 2 reveals a significant diminution in I_corr_ as the BM-01 concentration increased, whereas variations in E_corr_ showed no obvious trend. The E_corr_ of carbon steel increased from 18 to 34 mV in the presence of BM-01 assessed to the outgoing solution. According to certain theories, an inhibitor can be classified as “cathodic”, “anodic”, or “mixed” if its E_corr_ shift exceeds 85 mV from the E_corr_ of the reference solution^31^. If the offset is < 85 mV, the inhibitor is categorized as being of the mixed type.

This information suggests that BM-01 functioned as a mixed-type inhibitor and mostly displayed a cathodic action. Table 2 also shows variations in the anodic and cathodic Tafel slopes per concentration, with the cathodic slope showing a stronger trend. These results validated the dominant cathodic impact of BM-01 as a mixed-type inhibitor.

Table 2 clearly illustrates a substantial reduction in I_corr_ with increasing BM-01 concentration. For instance, I_corr_ decreased from 275.4 mA/cm^2^ in a HCl solution (1 M) to 27.54 mA/cm^2^ at 10^−3^ M of BM-01, thereby showcasing the inhibitory efficacy of BM-01. Alterations in the slope values of the anodic Tafel line (βa) in the presence of BM-01 suggested its adherence to the surface of the metal, occluding the activated sites and preventing the anodic process from taking place. Moreover, the modification of βc values upon BM-01 introduction further supported its inhibitory action.

#### EIS

We wished to deepen comprehension of the prevention of corrosion of carbon steel in challenging environments. We employed EIS to assess the impact of BM-01 at different concentrations on the interface between an aggressive solution and steel. EIS is a valuable method for evaluating corrosion inhibition because it provides insights into the: (i) electrical properties of the corrosion system; (ii) capacitance and resistance properties of the substrate (thereby enabling the precise inhibitory mechanism to be identified).

**Fig. 5:**
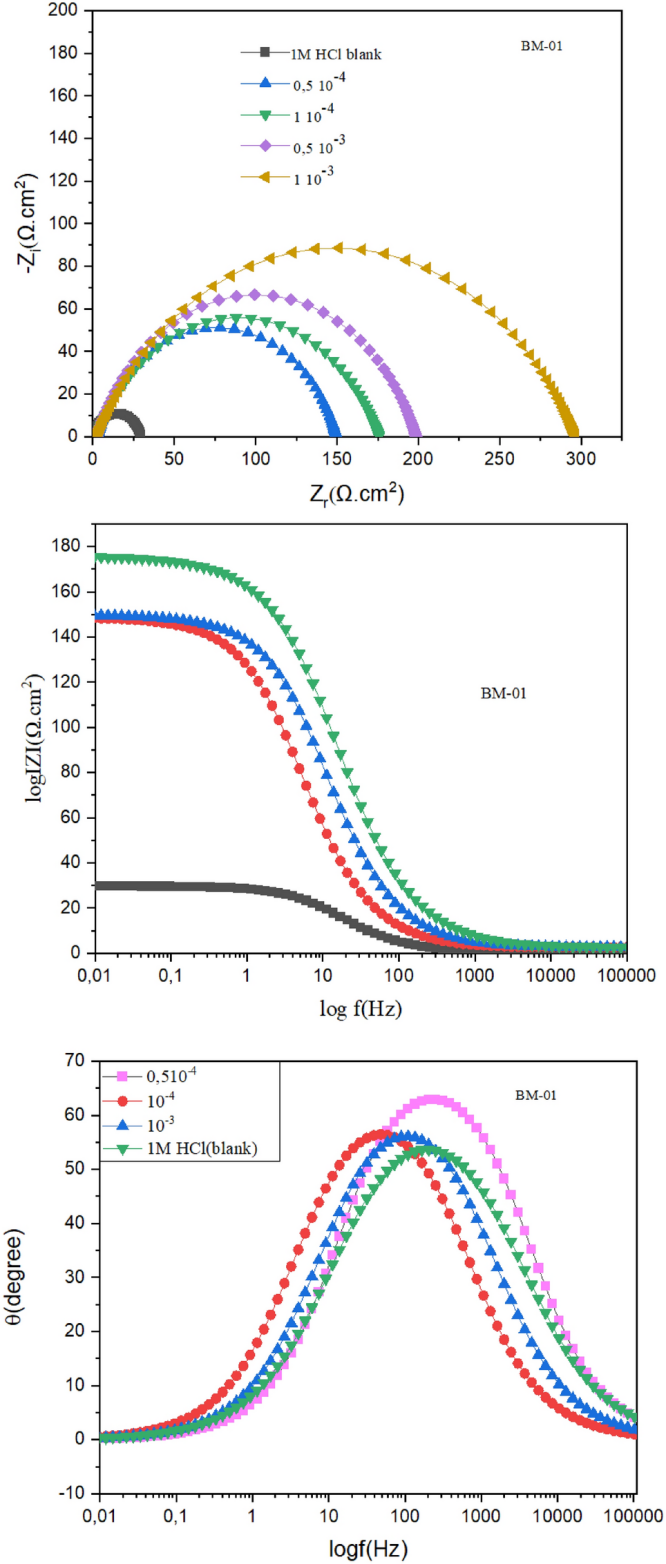
Bode Nyquist and phase angle diagrams for CS in 1.0 M HCl at different concentrations of BM-01 at 25 °C.

Nyquist plots were utilized to illustrate the impact of varying BM-01 concentrations of carbon steel submerged in a HCl solution (1 M) (Fig. 5). When BM-01 was absent, Nyquist plots exhibited a lone capacitive semicircle primarily at a high-frequency range. Upon the addition of BM-01 at different concentrations, noticeable changes occurred in the size and structure of Nyquist plots, particularly at lower frequencies^32^, where the emergence of an inductive loop became more pronounced^33^. The wider capacitive loop observed in Table 3 indicated that creation of a macromolecular matrix induced by BM-01 enhanced the resistance to corrosion by carbon steel.

**Table 3:** EIS parameters for C38 steel at 298 K in an uninhibited state and inhibited by several concentrations of BM-01.

Inhibitor	C (M)	RS (Ω cm^2^)	Rp (Ω cm^2^)	τdl (ms)	n	Cdl (μF cm^−2^)	η_*R*_ (%)
	Black	0.96	29.47	3.176	0.812	107.8	–
BM-01	0.5 × 10^−4^	2.68	146.2	13.792	0.691	94.34	79.86
	1.0 × 10^−4^	2.599	173.2	14.107	0.731	81.45	83.04
	0.5 × 10^−3^	2.373	196.3	15.028	0.763	76.56	85.04
	1 × 10^−3^	2.213	294.4	16.645	0.780	56.54	90.38

Figure S3 illustrates the characteristic equivalent electrical circuit (EEC) representing the adsorption of BM-01. To accommodate surface inhomogeneities, the double-layer capacitor (C_dl_) was substituted with the electrolyte resistor (R_s_), with a charge transfer resistor (R_P_) linked in parallel to a constant phase element (CPE)^34^.

### Adsorption isotherms

An inhibitor can be adsorbed on a metal surface. This action explains the increased inhibitory activity as the inhibitor concentration in an organic compound approaches that of the inhibitor. This phenomenon was responsible for the increase in inhibition efficacy observed in our study. However, these results may contradict the findings of Lorenz and Mansfeld^35^, but this phenomenon can be clarified by examining metal–inhibitor interactions^36^. Three inhibition modes can be observed at the interface; one of them involves the adsorbed inhibitor species physically blocking the surface of a metal electrode.

These species can adhere in two distinct manners: chemisorption and physisorption. Adsorption isotherms are utilized to obtain insights into the properties of the substances under examination. To generate an adsorption isotherm, the extent of coverage on the inhibitor surface must be determined.

We explored Temkin, Frumkin, and Langmuir adsorption isotherms (depicted in Figs. S4, S5, S6, and S7). The obtained data presented in Table 4 revealed that the Langmuir adsorption isotherm provided a comprehensive interpretation of the adsorption of the three types of inhibition, offering deep understanding of the adsorption process.

**Table 4:** Various parameters of adsorption-isotherm models for BM-01 on carbon steel at 298 K.

Model	Linear form	Curve	Parameter	Value
Langmuir^38^	$\frac{{C_{inh} }}{\theta } = \frac{1}{{K_{ads} }} + C_{inh}$	$\frac{{C_{inh} }}{\theta } = f\left( {C_{inh} } \right)$	R^2^	0.99963
			Slope	1.22941
			K_ads_ (M^−1^)	4.04E+04
			−ΔG_ads_ (kJ/mol)	33.55
			Standard deviation	1.33781E−5
Frumkin^39^	$$Ln\left( {C_{inh} .\frac{1 - \theta }{\theta }} \right) = - lnK_{ads} + 2*f*\theta$$	$$Ln(Cinh\left( {\frac{1 - \theta }{\theta } } \right) = f \left( \theta \right)$$	R^2^	0.71081
			Slope	7.4613
			Standard deviation	5.22076
Temkin^40^	$\theta = \frac{1}{f}lnK_{ads} + \frac{1}{f}C_{inh}$	$\theta = f\left( {lnCinh} \right)$	R^2^	0.93455
			Slope	5.45568
			Standard deviation	4.65789

The adsorption of inhibitors on metal surfaces was not well described by these models because the coefficients for the Temkin and Frumkin isotherms deviated greatly from the ideal^37^. These results confirmed that inhibitor adsorption on steel surfaces conformed to the Langmuir isotherm in HCl solution (1 M).

The standard free energy of adsorption (${\Delta G}_{{{\text{ads}}}}^{^\circ }$) and adsorption equilibrium constant (${\text{K}}_{{{\text{ads}}}}$) for an inhibitor molecule are linked by Eq. (10)^41^:

10 $$K_{ads} = \frac{1}{55,1}\exp \left( {\frac{{ - \Delta G_{ads}^{^\circ } }}{RT}} \right)$$

Table 5 provides an overview of the values for ${\text{K}}_{{{\text{ads}}}}$ and ${\Delta G}_{{{\text{ads}}}}^{^\circ }$. The perfect gas constant (R) is 8.314 J/mol K.

**Table 5:** Thermodynamic characteristics of adsorption of BM-01 on mild steel C38 in HCl (1 M) at different temperatures.

Inhibitor	T (K)	$K_{ads}^{^\circ }$(M^−1^)	$\Delta G_{ads}^{^\circ }$(kJ/mol)
BM-01	298	4.041E+04	− 33.55
	308	3.90E+04	− 23.35
	318	3.11E+04	− 24.50
	328	3.33E+04	− 25.94

In general, a high K_ads_ denotes efficient inhibition, implying a fast and efficient adsorption of the inhibitor onto a metal surface. N and π-electrons, two heteroatoms found in the BM-01 molecule, are responsible for the strong affinity between BM-01 and mild steel^42^.

Using the Gibbs–Helmholtz equation to calculate temperature-dependent variations in $\Delta G_{ads}^{^\circ }$, the standard entropy of adsorption (ΔS_ads_°) and standard enthalpy of adsorption (ΔH°_ads_) can be determined^43^ (Fig. S7).

11 $$\Delta G_{ads}^{^\circ } = \Delta H_{ads}^{^\circ } - T\Delta S_{ads}^{^\circ }$$

Strong and spontaneous contacts between inhibitor molecules and the metal surface are indicated by extremely negative values of $\Delta G_{ads}^{^\circ }$. If the value is close to or exceeds − 40 kJ/mol, then electron transfer begins, which creates a coordination bond for chemisorption between inhibitor molecules and the metal surface. In general, $\Delta {\text{H}}_{{{\text{ads}}}}^{^\circ }$ indicates interactions between charged molecules and metal charges via physisorption or electrostatic forces^44^.

The $\Delta {\text{H}}_{{{\text{ads}}}}^{^\circ }$ obtained in our study for BM-01 was − 20 kJ/mol in HCl. This result implied that primarily physical adsorption occurred on the surface of mild steel (Table 6).

**Table 6:** Aspects of carbon-steel activation in HCl (1 M) with various concentrations of BM-01.

Inhibitor	C (M)	$E_{a}$(kJ/mol)	∆Ha (kJ/mol)	− ∆Sa (J/mol)	*Ea* − *ΔH*^*°*^*a* (kJ/mol)
	Black	45.04	42.45	95.6316	2.59
BM-01	0.5 × 10^−4^	49.82	47.23	89.4631	2.59
	10^−4^	52.22	49.63	85.7252	2.59
	0.5 × 10^−3^	52.78	50.19	84.0008	2.59
	10^−3^	64.34	61.74	50.791	2.60

### Determination of the activation energy (E_a_)

In most cases, an increase in temperature accelerates processes and, in this case, increased the rate of corrosion considerably.

In practice, for example, chemical pickling of steels in acid baths is carried out at high temperatures. In this process, inhibitors are incorporated naturally to protect the metal surface. Researchers have investigated the impact of temperature on the stability and efficacy of inhibitors derived from plants or organic sources at various temperatures.

The Arrhenius equation is used to describe the influence of temperature on the corrosion rate:

12 $$W_{corr} = A\exp \left( { - \frac{Ea}{{R*T}}} \right)$$

where T is the absolute temperature in Kelvin (K), R is the ideal gas constant, A is a constant factor, and W_corr_ is the corrosion rate.

E_a_ can be visually determined using Eq. (12) in its logarithmic form^45^:

13 $$\ln \left( {W_{corr} } \right) = \ln A - \frac{Ea}{{R*T}}$$

Enthalpy is determined using Eq. (14)^44^:

14 $${\text{Ln}}\left( {\frac{{\text{w}}}{{\text{T}}}} \right) = \left( {\ln \left( {\frac{{\text{R}}}{{{\text{N}}_{{\text{a}}} *{\text{h}}}}} \right) + \frac{{\Delta {\text{S}}_{{\text{a}}}^{^\circ } }}{{\text{R}}}} \right) - \frac{{\Delta {\text{H}}_{{\text{a}}}^{^\circ } }}{{{\text{R}}*{\text{T}}}}$$

where $\left( {ln\left( {\frac{R}{{N_{a} *h}}} \right) + \frac{{\Delta S_{a}^{^\circ } }}{R}} \right)$ is a constant and ∆H°_a_ is the enthalpy of activation.

Measurements of weight loss were conducted over a range of temperatures (298–328 K) with and without BM-01. This was undertaken to elucidate the inhibition mechanism and determine the E_a_ associated with the corrosion process.

The graphical representation of $ln\left( {W_{corr} } \right)$ as a function of (1000/T) yielded straight lines (Fig. S8) with a slope of $\left( { - \frac{Ea}{{R*T}} } \right)$. Hence, E_a_ was calculated.

Similarly, Fig. S8 illustrates the linear relationship obtained by plotting ln(W_corr/T) against (1000/T). The calculated values of E_a_, ΔH°_a_, and ΔS°_a_ are shown in Table 6.

The data in Table 6 show that samples containing BM-01 exhibited higher values of E_a_ compared with samples not containing BM-01. Typically, an increase in E_a_ is associated with physical adsorption^46,47^. This phenomenon could be due to inhibitor molecules having higher mobility at higher temperatures, allowing them to avoid adsorption onto the steel surface. That is, at higher temperatures, the balance between the adsorption and desorption of inhibitor molecules leans towards desorption. More contact between corrosive media and the metal surface would be produced by increased desorption of BM-01 molecules from the steel surface. Consequently, at increased temperatures, the corrosion rate increases^48,49^.

The estimates of E_a_ in our study (45.04–66.95 kJ mol^−1^) are notably higher when compared with the chemisorption threshold of 80 kJ mol^−1^^50^.

∆H_a_ provides further insights into the corrosion process^51^. The process of steel dissolution is endothermic (as indicated by the positive sign of ∆H°_a_) An increase in ∆H°_a_ suggests a potential reduction in the rate of steel corrosion. Increased and negative values of ΔS°_a_ suggest a decrease in disorder when reactants transform into the activated iron–molecule complex in solution^52^.

In parallel, the rise in ∆H_a_ suggested a possible reduction in the corrosion rate of steel. As the reactants transformed into the solution-activated iron–molecule complex, the high, negative values of ΔS°_a_ indicated a reduction in disorder.

In HCl (1 M), the average difference between Ea and ∆H°_a_ was 2.59 kJ mol^−1^, closely resembling the room-temperature value of 2.60 kJ mol^−1^. This observation suggested that ∆H°_a_ and E_a_ exhibited similar variations upon BM-01 addition, and that they were related by Eq. (15)^53^:

15 $$Ea - \Delta H_{a}^{^\circ } = RT$$

### EDS and SEM

Utilizing high-resolution SEM, we analyzed the surface structure of C38 steel and understood the effects of inhibitors upon corrosion. These images were captured by immersing steel plates in an acid solution for 12 h with and without inhibitors. Then, EDS was carried out to determine the fundamental composition of the steel surface^54^. EDS and images of samples of C38 steel are depicted in Fig. S11 under two conditions: (a) no inhibitor was present in HCl solution (1 M); (b) BM-01 (10^−3^ M) was present in HCl solution (1 M).

The emergence of dark patches on the steel surface (Fig. S9a) clearly indicated that the material has suffered significant damage. EDS revealed an oxygen peak, and confirmed that these spots represented widespread corrosion of steel. This peak suggested that, throughout the corrosion process, the steel may have undergone oxidation.

Figure S9b depicts a distinct surface morphology from Fig. S9a, without the presence of black patches indicative of corrosion. This observation testified to the effectiveness of BM-01 in providing protection to the steel surface by creating a barrier between the corrosive environment and metal surface. This inhibitory layer delayed corrosion. In comparison with Fig. S9a, the EDS of Fig. S9b exhibited fewer oxygen peaks.

### DFT calculations for the examination of electronic structure

DFT calculation is a highly suitable method for assessing the reactivity of BM-01. The parameters ΔE_gap_, E_HOMO_, ΔN_(110)_, and µ reflect the efficacy of inhibition of a compound, such as BM-01, applied to the surface of carbon steel^55^.

Acidic conditions are characterized by an excess of protons, which enhances the probability of protonation of organic molecules. The precise location for protonation was the nitrogen atom N3 which, with a pH of 4.87, was the only active site for binding H^+^ (Fig. S10). These outcomes were obtained using MarvinView software^56^.

Figure S13 illustrates the delivery of electrostatic potentials and optimal configurations of BM-1. These configurations signify the highest stable states with the lowly energy. Notably, the Molecular electrostatic potential (MEP) distribution highlighted high density at N3 nitrogen atoms and the phenyl ring connected to nitrogen atom N6 (red highlighting in Fig. S13). This arrangement facilitates the sharing of electron density with vacant iron orbitals^57^. Upon protonation to form BM-01(+1), the molecular surface structure appeared blue, indicating reduced electron density. This finding suggested more cationic behavior and lower reactivity to chemicals, thus reducing its electron-transfer capacity.

**Fig. 6:**
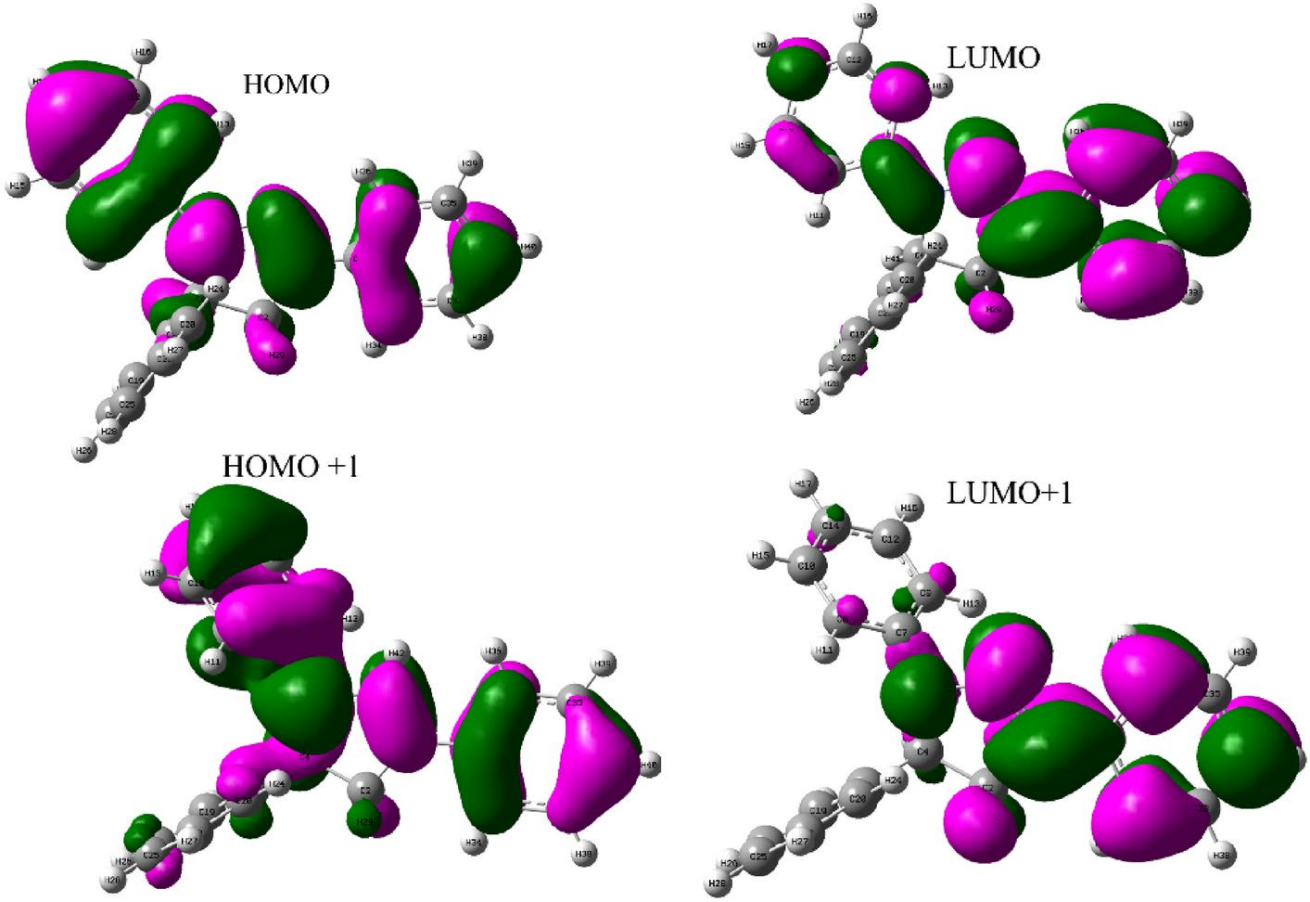
FMO distributions for neutral (BM-01) and protonated (BM-01 (+ 1)) forms.

Overall reactivity is governed by the distribution of electron density within LUMO and HOMO molecular states. In BM-01 and BM-01(+1) forms, the electron density in the HOMO covered the entire chemical surface (Fig. 6) with the exception of phenyl carbon atoms (which were bound to C4). This finding highlighted that BM-01 and BM-01(+1) had numerous active electron-donor sites distributed over their entire molecular structure. This observation emphasizes the importance of adhesion between BM-01 and BM-01(+1), respectively, and the metal surface. The density distributions of HOMO and LUMO remained essentially unaltered, indicating that protonation did not affect their density distribution.

Table 7 summarizes the chemical structural characteristics for protonated and neutral forms. The significant chemical reactivity of BM-01 with the metal was highlighted by its higher E_HOMO_ (− 5.2808 eV) in the neutral state, which showed that it could donate electrons readily to unoccupied orbitals on the metal surface^58^. E_HOMO_ decreased after protonation (− 6.817 eV) compared with that obtained with neutral BM-01, indicating that the electron-donating capacity had been reduced (∆N = 0.5474). This result showed that BM-01 in the protonated state was less active than in the neutral state.

**Table 7:** Quantum mechanical characteristics of BM-01.

Inhibitor	E_homo_ (ev)	E_lumo_ (ev)	∆E (eV)	µ (Debye)	η (eV)	σ (eV^−1^)	χ	ΔN
BM-01	− 5.2808	− 1.5248	3.7560	3.850	1.878	0.532	3.403	0.9577
BM-01(+)	− 6.817	− 2.9718	3.8458	6.410	1.923	0.520	4.895	0.5474

BM-01 was poised to distribute its electrons, as evidenced by its positive ΔN value in the neutral state, which was < 3.6, data which are in accordance with the findings of Lukovit^59^. This electron-sharing behavior stemmed from the presence of active centers in BM-01; specifically, the nitrogen atom (N3) and carbon atoms (C37), as well as the electron cloud of the associated aromatic ring. E_LUMO_ became more negative after protonation, suggesting a decrease in inhibition capacity. Therefore, the energy gap (ΔE_gap_) between E_HOMO_ and E_LUMO_ represents chemical reactivity; a large value indicates that the molecule under investigation is less reactive toward iron atoms. Two other parameters of crucial importance are overall hardness (η) and overall softness (σ). The latter is the reciprocal of hardness (1/η). σ is also quite important if inhibitor molecules adhere to the iron surface^27^. Table 7 reveals that the calculated values for σ increased, confirming once again that the inhibition efficacy followed the expected order BM-01 > BM-01(+). This observation implied that the neutral state of BM-01 worked better than the protonated form.

### Fukui indices

Local reactivity is a commonly used method for identifying local active sites within an inhibitor molecule.

Fukui parameters were used to identify the optimal adsorption sites in the optimized structure of the BM-01 molecule. Table S1 shows the results of exploiting the local molecular reactive properties of BM-01.

The most nucleophilic ($f_{k}^{ - }$) and electrophilic ($f_{k}^{ + }$) attack sites of the BM-01 molecule were measured using the Fukui function. The Fukui analysis was conducted using natural population analysis. Then, the Fukui indices ($f_{k}^{ - } ,f_{k}^{ + }$) of the BM-01 molecule were calculated using the Fukui functions, which are described in Eqs. 16 and 17^60^.

16 $$f_{k}^{ + } = q\left( {N + 1} \right) - q\left( N \right)$$

17 $$f_{k}^{ - } = q\left( N \right) - q\left( {N - 1} \right)$$

The parameters of local reactivity, such as Fukui’s double descriptors $f_{k}^{2}$ (which represent the difference between Fukui’s nucleophilic and electrophilic functions) are more accurate and consistent tools than the indices of local reactivity mentioned above. Moreover, $f_{k}^{2}$ is calculated according to Eq. (18).

18 $$f_{k}^{2} = f_{k}^{ + } - f_{k}^{ - }$$

Note that the process is favored for electrophilic attack if the local double descriptors $f_{k}^{2}$ are < 0, while nucleophilic attack is favored if $f_{k}^{2}$ > 0^61^.

The BM-01 molecule had several electrophilic and nucleophilic active sites, which could favor its adsorption onto an iron surface (Table S1). In accordance with quantum descriptors, the Fukui index highlighted that nitrogen atoms and some carbon atoms acted as nucleophilic sites. They provided the electrons needed to create a coordination connection with the molecular orbital of the metal surface. However, other carbon atoms were electrophilic sites, accepting electrons from the surface.

**Fig. 7:**
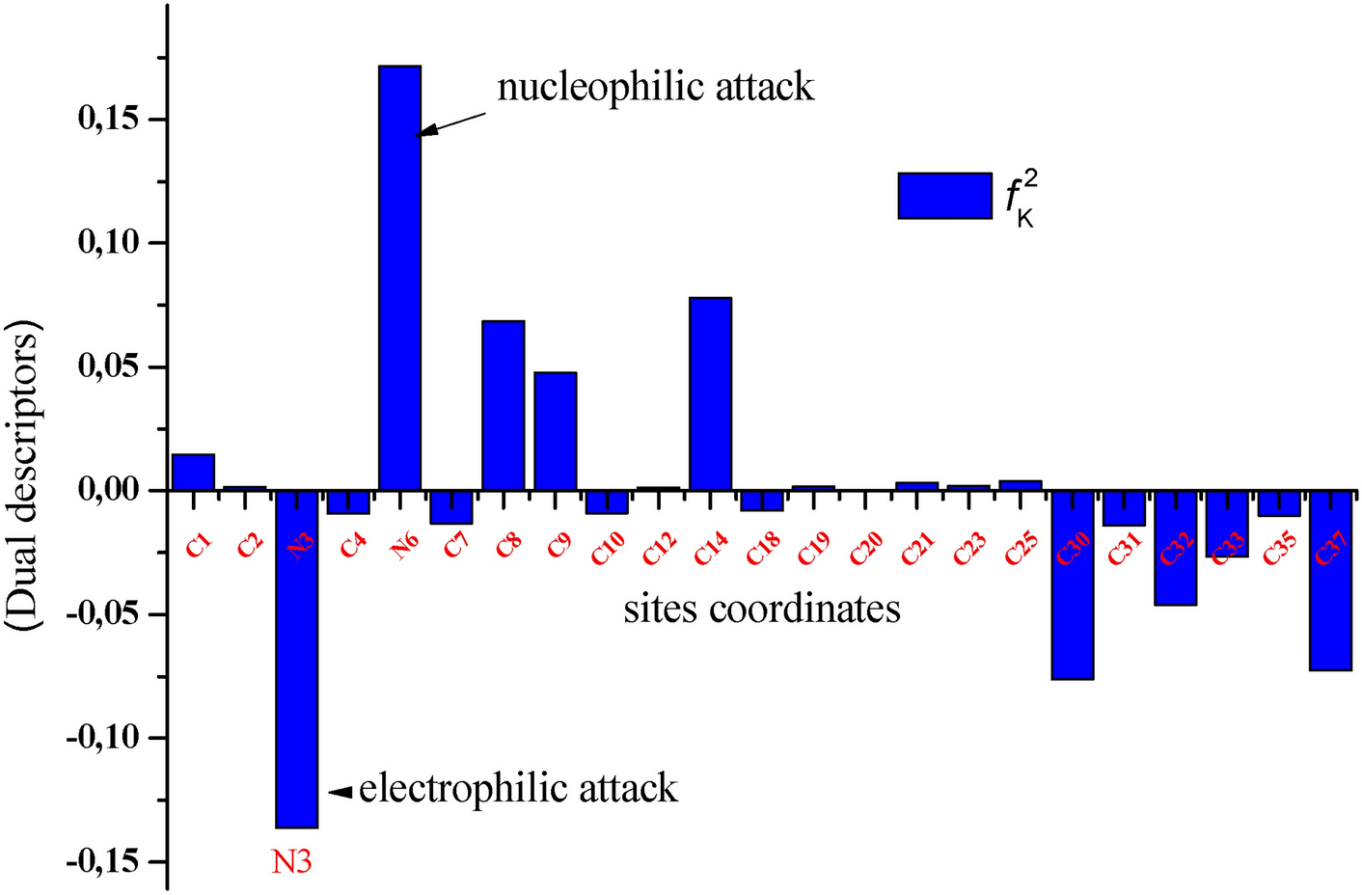
Graphical representation of the double descriptors ($f_{k}^{2}$) for the most active BM-01 sites studied using the DFT method with the B3LYP/6-311G(d,p) base set.

Fukui’s double descriptors ($f_{k}^{2}$) for the most active sites of BM-01 are shown in Fig. 7, this approach shows promise in predicting reactivity and detecting issues with regioselectivity^62^. The sequence of the nucleophilic sites was N3 > C30 > C37 > C32 > C31. Conversely, N6, C8, and C14 were electrophilic sites.

### Sites favored for protonation

The proton affinity (PA) measures the permanence of a compound to pinpoint optimal protonation sites for a compound. We aimed to determine appropriate protonation of BM-01 employing Eq. (19):

19 $$PA = E_{prot} + E_{H2O} - \left( {E_{neutral} + E_{{H3O^{ + } }} } \right)$$

where E_prot_ and E_neutral_ represent the full energy of protonated and non-protonated inhibitors, respectively. The total energy of the molecule, E_H2O_, equates to the complete energy of the hydronium ion, E_H3O_+.

We examined the protonation of two nitrogen sites: N3 and N6. Calculations showed that the protonation followed an exothermic process, suggesting that our inhibitors tended to be protonated. Furthermore, research has shown that the more negative the PA, the greater is the inhibitory effect because PA is related to basicity^63^. The PA at N3 and N6 were − 442.9650 kcal/mol and − 439.2992 kcal/mol, respectively, which suggested a preference for protonation at N3 over N6. This result is consistent with local and global electronic characteristics, as well as with MarvinView results.

#### Electron localization function (ELF)

**Fig. 8:**
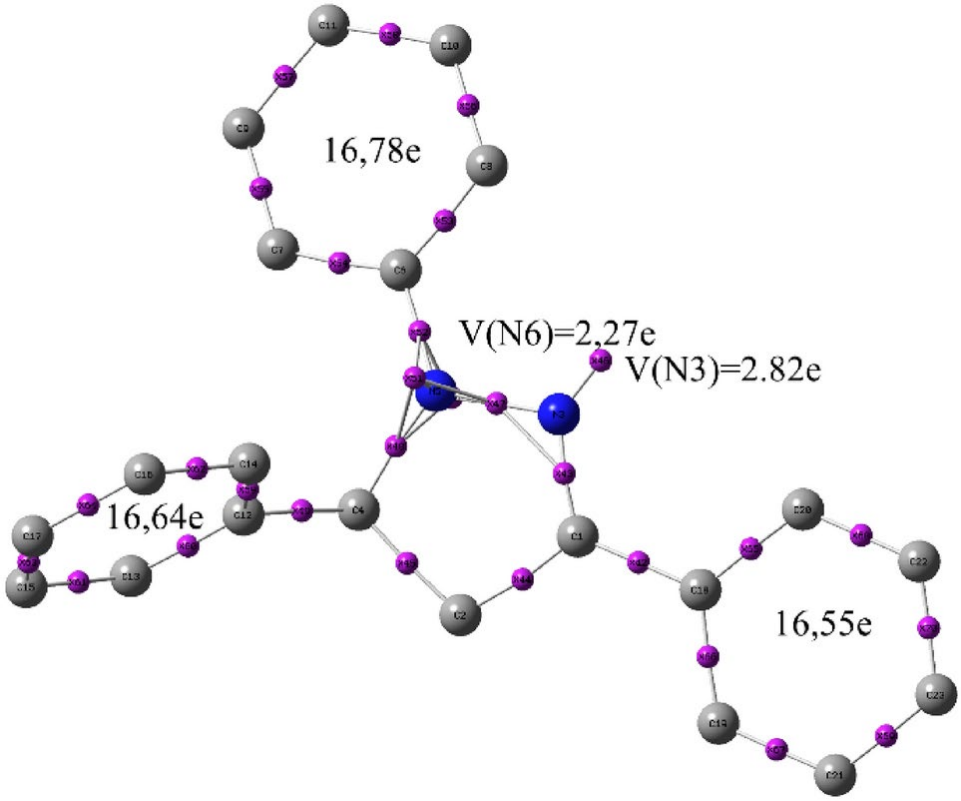
Populations of the ELF valence basins, in e, of BM-01.

Innovatively, when examining the inhibition of corrosion, one absolute bond between a chemical structure and electron-density distributions is provided by the ELF generated through quantum topological analysis. ELF also identifies the binding and non-binding regions present in a chemical system, Monosynaptic V(X) pools correspond to non-binding regions in the context of this approach, while di-synaptic V(X, Y) pools are linked to binding regions^64^. ELF analysis of this structure was carried out using Multiwfn (Fig. 8).

ELF analysis of BM-01 revealed many monosynaptic pools bound to the N3 atom (V(N_3_) = 2. 82e). Hence, the phenyl ring bound to the N3 nitrogen atom had a large total bi-synaptic electron population of ~ 16.75 e compared with that of the other two phenyl rings. This result implied that the molecule contained a fairly large electron population, which tended to give an electron density of metallic iron. This finding was consistent with results after calculation of global and local indices (Figs. 6 and 7).

#### Electronic characteristics of Fe–(BM-01)

To assess how the iron surface affects the electron-density distribution and quantum chemical descriptors of BM-01, B3LYP/LanL2DZ DFT simulations were conducted for the Fe–BM-01 complex. Gaussian 09 was employed for the computation of chemical quantum properties^23,57^.

For the Fe–(BM-01) complex, Fig. S12 illustrates the electron-density distribution and optimized structure of molecular frontier orbitals (FMOs). When BM-01 was coordinated with the iron atom, its optimal spatial conformation remained relatively unchanged from its individual structure. Table 8 lists the key descriptors in quantum chemistry. These data revealed that the iron atom enhanced the overall chemical reactivity. This was evident in the increased dipole moment and ΔN, along with a decrease in ΔE_gap_. These observations suggested that BM-01, if attached to the iron atom, exhibited effective reactivity with the surface. Given the properties of the environment, the adsorption mechanism at the organic–metal surface was examined using MD simulations^58^.

**Table 8:** Chemical structural descriptors of the Fe–BM-01 complex.

Complex	E_HOMO_ (ev)	E_LUMO_ (ev)	∆E (eV)	µ (Debye)	ΔN
Fe–(BM-01)	− 5.280	− 0.603	4.677	9.220	0.8676

In the neutral state, BM-01 adsorbed in the planar direction at 298 K on the surface of carbon steel in an acidic solution, this adsorption was attributed to the covalent bonds formed at the interface between BM-01 and the Fe (110) surface. BM-01 showed reduced adsorption at a higher temperature (328 K). The interaction capacity between BM-01 and the metal was evidenced by the negative values of the computed interaction energy. At 298 K, this interaction energy was − 344.254 kcal/mol, and at 328 K, it was − 341.435 kcal/mol. These findings indicated that as the temperatures increased, the efficacy of BM-01 in interacting with iron atoms on the surface diminished.

**Fig. 9:**
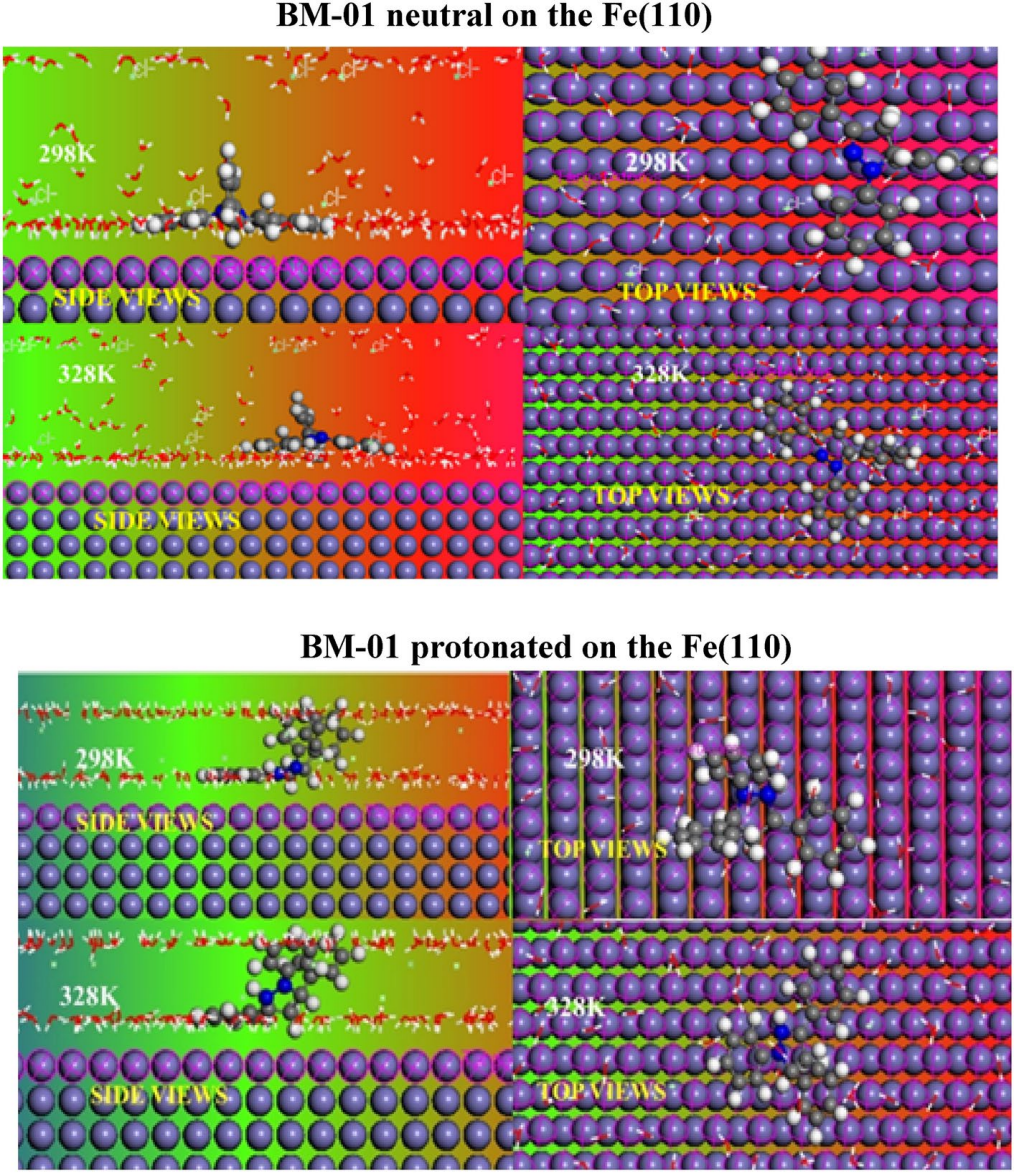
More stable configurations of neutral and protoned BM-01 on the Fe(110) surface at T = 298Kand T = 328 K using the MD method.

The adsorption of BM-01(+) on the iron surface in an acidic solution is shown in Fig. 9, illustrating side and top views, respectively, at two simulated temperatures. These observations indicated structural modifications in BM-01(+) that reduced surface adsorption compared with that of BM-01. The estimated interaction energy of the protonated inhibitor was − 336.043 kcal/mol for 298 K and − 316.234 kcal/mol for 328 K. This phenomenon was likely due to the physical connection among adsorbed chlorine ions on the surface of the metal and protonated nitrogen atom N3. This bond, subjected to temperature modifications, explains the reduced efficiency of BM-01(+) compared with that of BM-01.

#### Radial distribution function (RDF)

RDF, represented as g(r), is a commonly employed method for assessing the proximity as well as the interaction between inhibitor molecules and a metal surface. The RDF of chemisorption is, in general, maximal between 1 Å and 3.5 Å, whereas physisorption is marked by peaks at > 3.5 Å^65^. By MD simulations at T = 298 K and T = 328 K, the g(r) function was examined with regard to most activated atom, N3 of BM-01, in relation to iron atoms on the surface.

Figure S13 reveals that, at T = 298 K, the minimum distance between the N3 atom and Fe (110) surface was ~ 2.73 Å. This finding suggested that predominant chemical interactions occurred between iron atoms and the inhibitor molecule, which could promote the adsorption of BM-01 and, thus, provide enhanced protection of iron against corrosion. As the temperature increased to T = 328 K, the interatomic distance increased, thereby reducing the adsorption efficiency^49^.

#### Inhibitor/water/Fe_2_O_3_ (110) systems

**Fig. 10:**
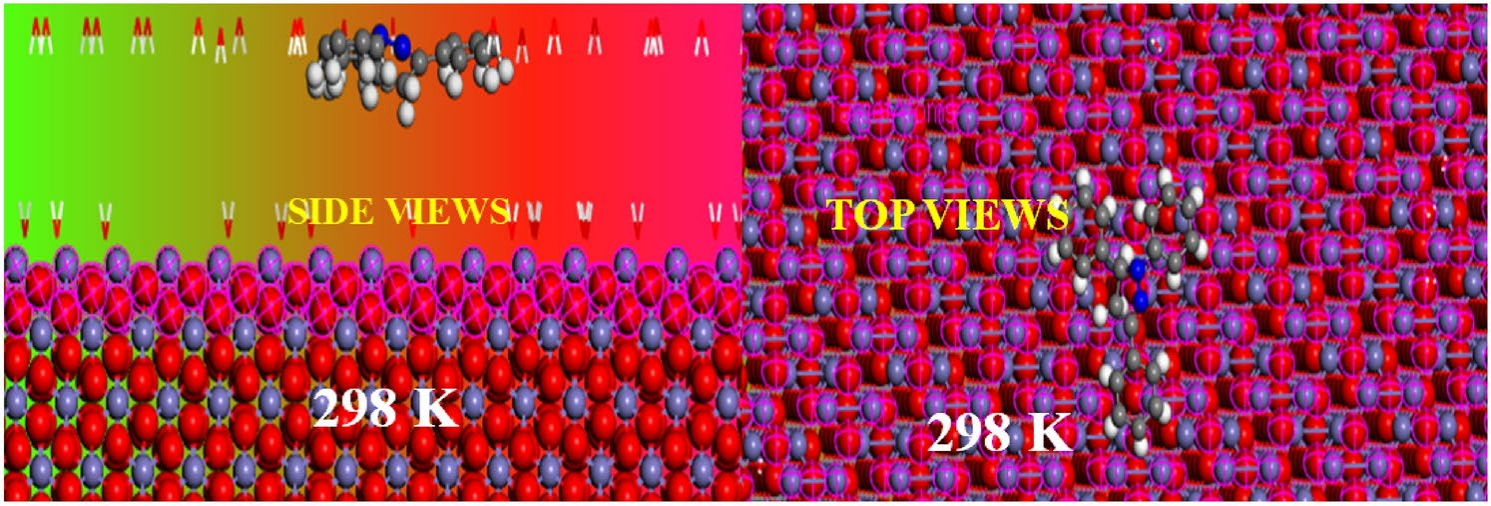
Equilibrium adsorption modes of the inhibitor molecule on the Fe_2_O_3_ surface (110).

The decision to focus on the Fe_2_O_3_ surface was motivated by the risk of prior oxidation of metallic iron in the presence of an acidic solution. Iron oxides such as Fe_2_O_3_ can form on the surface of carbon steel in an acidic environment, we simulated the adsorption of BM-01 on the Fe_2_O_3_ (110) surface. The configurations obtained reveal that BM-01 enveloped the Fe_2_O_3_ (110) surface partially, with lateral adsorption on the Fe_2_O_3_ surface (Fig. 10), while Table 9 provides energy results and descriptors (in kcal/mol) obtained from Monte Carlo simulations of BM-01 on Fe (110) and Fe_2_O_3_ (110).

**Table 9:** Energy results and descriptors (in kcal/mol) obtained by Monte Carlo simulations of BM-01 on Fe (110) and Fe_2_O_3_ (110).

Structures	Total energy	Adsorption energy	Rigid adsorption energy	Deformation energy	MB01H : dEad/dNi	H20 : dEad/dNi
Fe (1 1 0)	− 6.21E+04	− 6.21E+04	− 7.91E+04	1.69E+04	− 662.557	− 534.437
Fe_2_O_3_ (1 1 0)	− 2.17E+03	− 2.15E+03	− 2.15E+03	3.9028929	− 167.852	− 7.51869

## Conclusions

The synthesis and characterization of the pyrazole-derived molecule BM-01 were conducted using spectroscopy (^1^H, ^13^C NMR, ATR-IR). Subsequently, its corrosion inhibition on carbon steel was assessed using a HCl solution (1 M). Evaluations involved various methods (electrochemical, weight loss, computational).

BM-01 prevented steel corrosion effectively. Its inhibitory efficacy increased proportionally with concentration to ~ 90.38%. These findings aligned with those obtained through other methods (PDP, electrochemical impedance, weight loss). BM-01 was a mixed-type inhibitor, predominantly exhibiting cathodic efficacy, as indicated by Tafel slopes.

A structural model was postulated using EIS. Adsorption of BM-01 on a steel surface inhibited corrosion, and aligned with Langmuir’s thermodynamic model. The predominant adsorption mechanism for BM-01 was chemisorption. SEM and EDS revealed a protective coating on the surface. Theoretical predictions from DFT calculations and MD simulations closely mirrored experimental observations.

## Supplementary Information

Supplementary Information.

## Data Availability

All data generated or analysed during this study are included in this published article [and its supplementary information files].

## Code availability

The calculations were performed using Gaussian 09 software provided by Gaussian, BIOVIA Discovery and Studio Visualizer provided by BIOVIA.
