# Supplementary material for: Design, synthesis, and evaluation of a pyrazole-based corrosion inhibitor: a computational and experimental study
Source: Sci Rep. 2024 Oct 24;14:25238. doi: 10.1038/s41598-024-76300-5 (PMC11502868; doi:10.1038/s41598-024-76300-5)
Supplement: Supplementary file 1 — Supplementary Information. [file 41598_2024_76300_MOESM1_ESM.docx]

**Design, Synthesis, and Evaluation of a Pyrazole-based Corrosion Inhibitor: a Computational and Experimental Study**

Abdelmalek Matine^1^, Bouchra Es-Sounni^2^, Mohamed Bakhouch^2^, Ali H. Bahkali ^3^, Habib El Alaoui El Abdallaoui^1^, Shifa Wang^4^, Asad Syed^3^, Ling Shing Wong^5^, Na'il Saleh^6*^, Abdellah Zeroual^1*^

^1^ Molecular Modeling and Spectroscopy Research Team, Faculty of Sciences, Chouaib Doukkali University, PB 20, 24000 El Jadida, Morocco

^2^ Bioorganic Chemistry Team, Department of Chemistry, Faculty of Sciences, Chouaïb Doukkali University, P.O. Box 24, 24000 El Jadida, Morocco.

*^3^ Department of Botany and Microbiology, College of Science, King Saud University, Riyadh, Saudi Arabia*

*^4^ School of Electronic and Information Engineering, Chongqing Three Gorges University, Chongqing, Wanzhou, 404000, China*

*^5^ Faculty of Health and Life Sciences, INTI International University, Putra Nilai, 71800 Nilai, Negeri Sembilan, Malaysia.*

*^6^ Department of Chemistry, College of Science, United Arab Emirates University, Al Ain P.O. Box 15551, United Arab Emirates.*

**Corresponding author:* [*n.saleh@uaeu.ac.ae*](mailto:n.saleh@uaeu.ac.ae)*, zeroualabdellah2@gmail.com*

| **Content** | **Pages** |
| --- | --- |
| Figure S1*.* Synthetic pathway of the studied pyrazole BM-01. | S-2 |
| Figure S2. Infrared spectrum of compound BM-01 | S-2 |
| Figure S3. Equivalent electrical circuit used for impedance spectrum obtained for BM-01 in 1M | S-2 |
| Figure S4. Langmuir adsorption isotherm for C48 carbon steel in HCl (1M) in the presence of BM-01 at different temperatures | S-3 |
| Figure S5. Temkin adsorption isotherm for BM-01 on C38 mild steel in 1M HCl at 298K | S-3 |
| Figure S6. Frumkin adsorption isotherm for BM-01 on C38 mild steel in 1M HCl at 298K | S-4 |
| Figure S7. Temkin adsorption isotherm for BM-01 on C38 mild steel in 1M HCl at 298K | S-4 |
| Figure S8. Variation $ln(W_{corr})$ as a function of $(\frac{1000}{T})$ of the corrosive medium in the absence and presence of BM-01 | S-5 |
| Figure S9. SEM and EDS spectrum images of carbon steel: (a) immersion in 1 M HCl (b) immersion in 10^-3^M BM-01 | S-5 |
| Figure S10. Sites favorable to protonation in an acid medium | S-6 |
| Figure S11. Optimized structures and distribution of neutral (BM-01) and protonated (BM-01 (+1)) PEMs | S-6 |
| Table S1. Fukui functions, dual Fukui functions for BM-01 | S-7 |
| Figure S12. FMO distributions of the Fe-BM-01 complex | S-7 |
| Figure S13. FDRs of the Fe (1 1 0) surface relative to the N3 atom of the neutral BM-01 at 298 and 328 K | S-8 |

**Figure S1*.*** Synthetic pathway of the studied pyrazole BM-01.

**
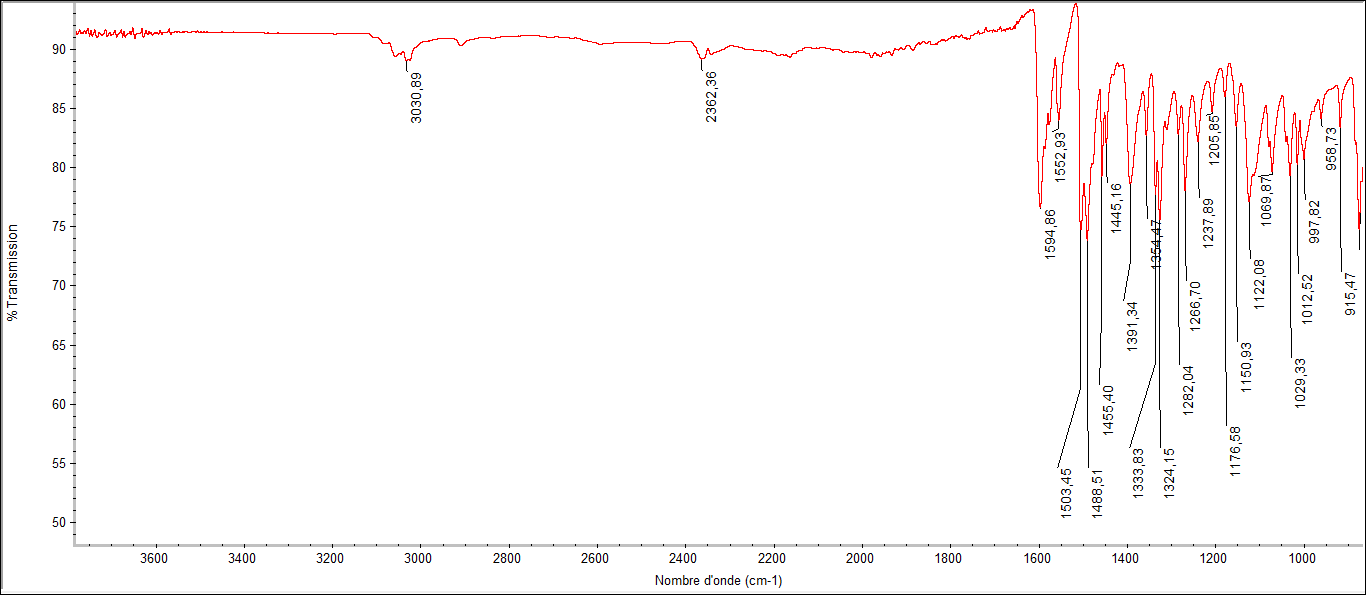
**

**Figure S2. Infrared spectrum of compound BM-01**

**
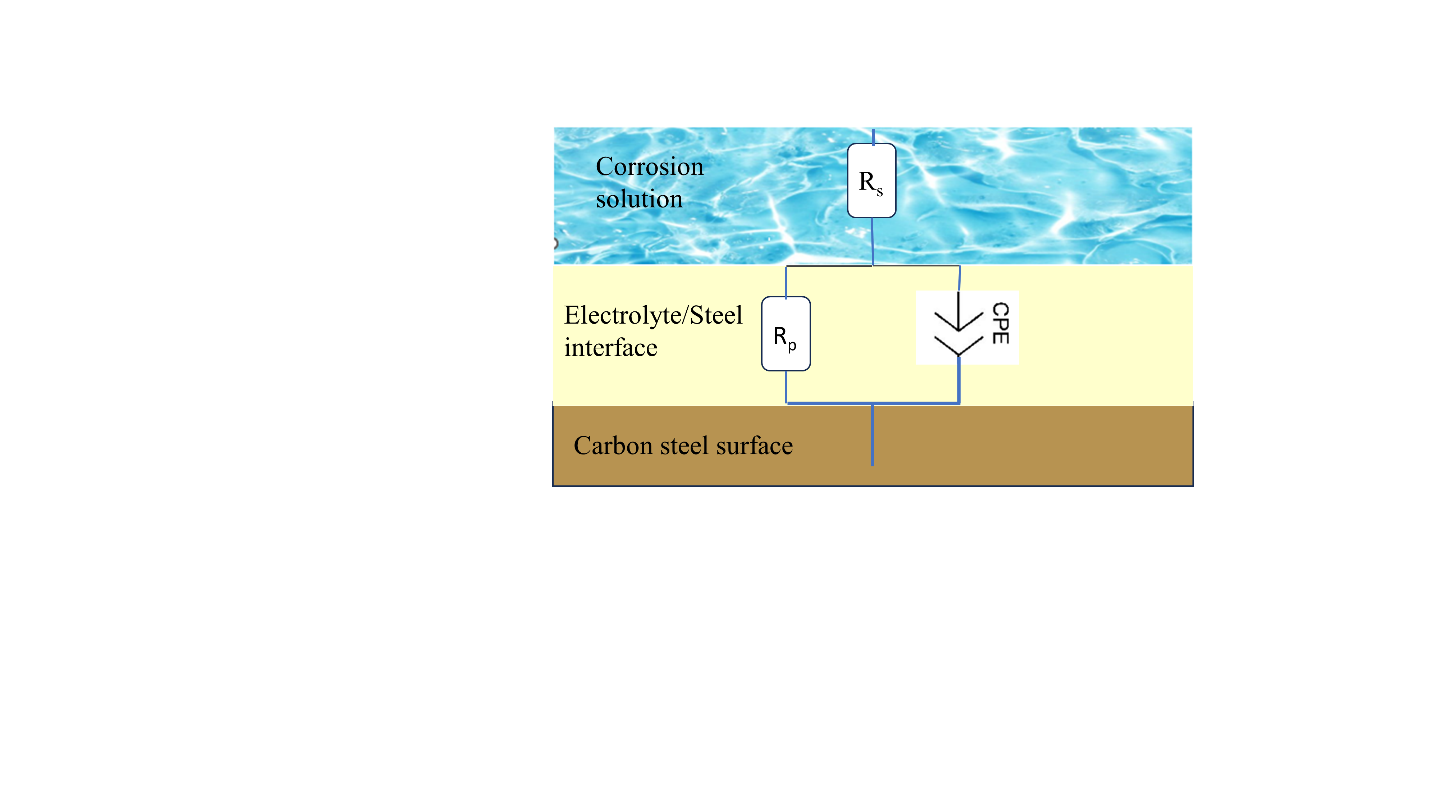
**

**Figure S3.** Equivalent electrical circuit used for impedance spectrum obtained for BM-01 in 1M


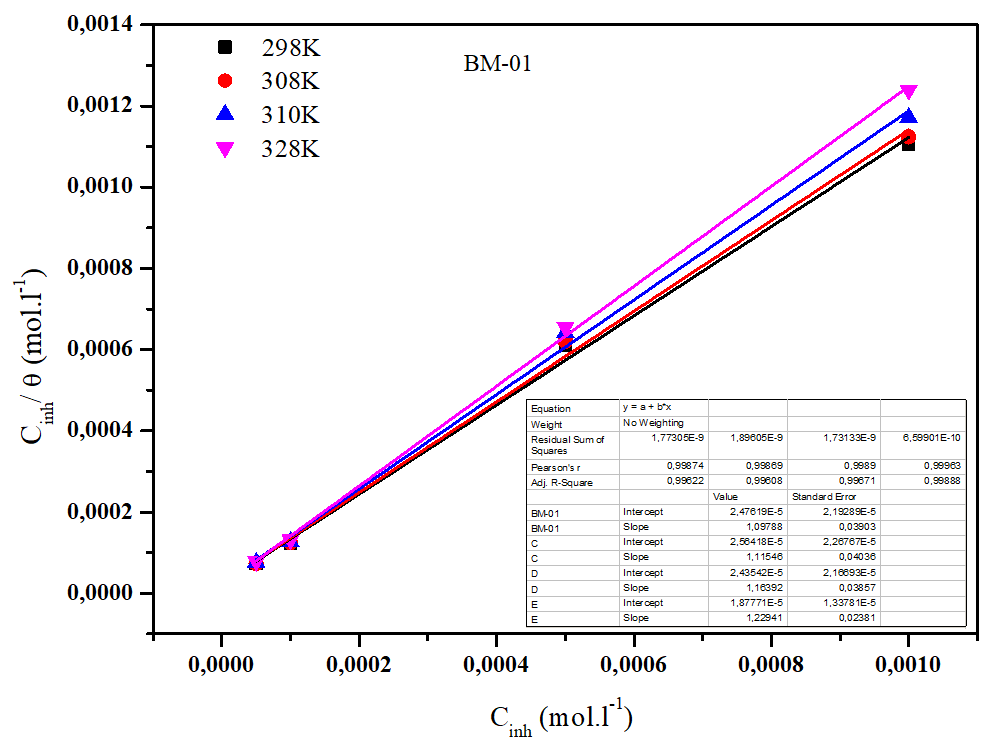


**Figure S4.** Langmuir adsorption isotherm for C48 carbon steel in HCl (1M) in the presence of BM-01 at different temperatures


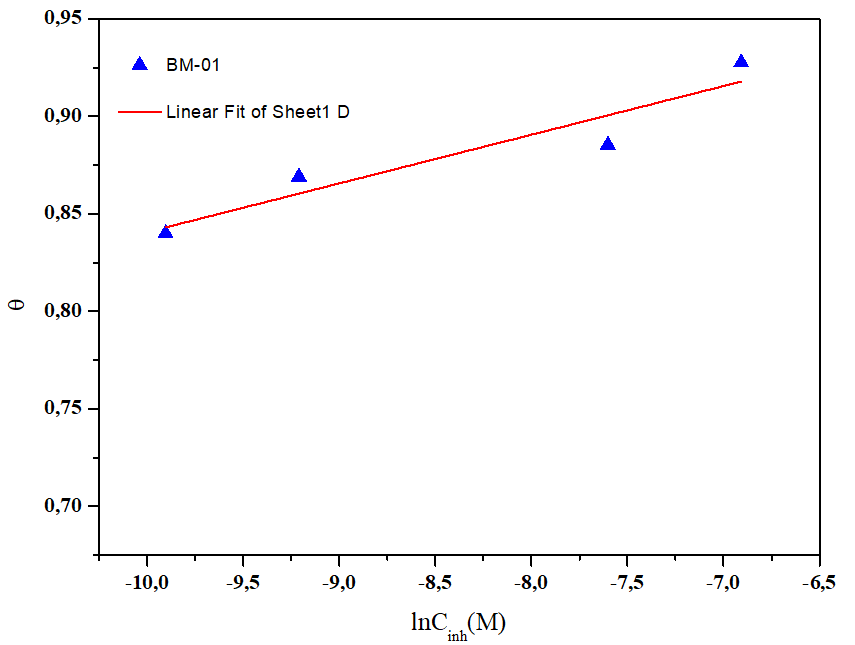


**Figure S5**. Temkin adsorption isotherm for BM-01 on C38 mild steel in 1M HCl at 298K


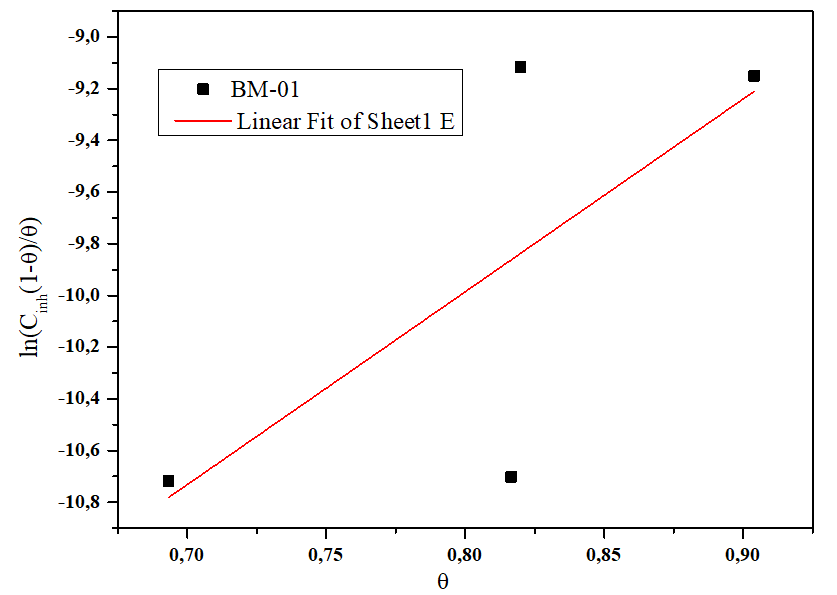


**Figure S6.** Frumkin adsorption isotherm for BM-01 on C38 mild steel in 1M HCl at 298K


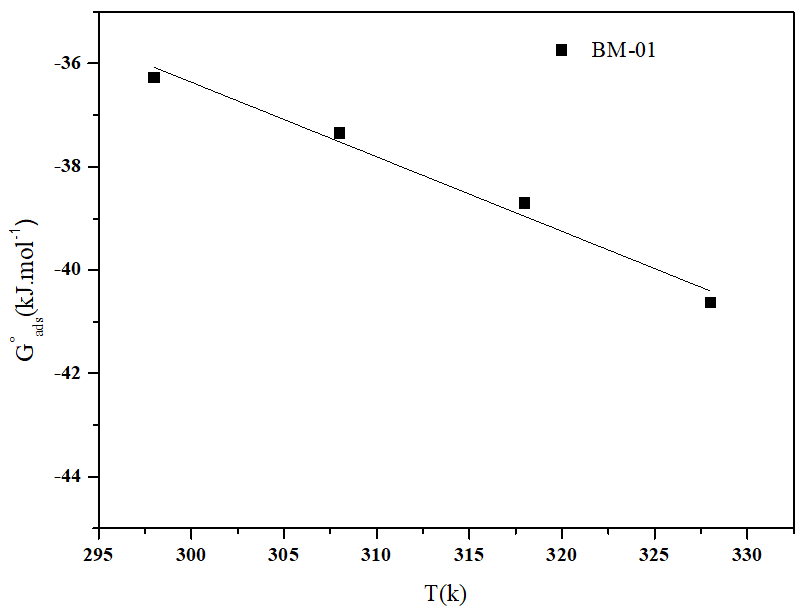


**Figure S7**. Temkin adsorption isotherm for BM-01 on C38 mild steel in 1M HCl at 298K

**Figure S8. Variation** $\boldsymbol{ln(}\boldsymbol{W}_{\boldsymbol{corr}}\boldsymbol{)}$ **as a function of** $\boldsymbol{(}\frac{\boldsymbol{1000}}{\boldsymbol{T}}\boldsymbol{)}$ **of the corrosive medium in the absence and presence of BM-01**

**
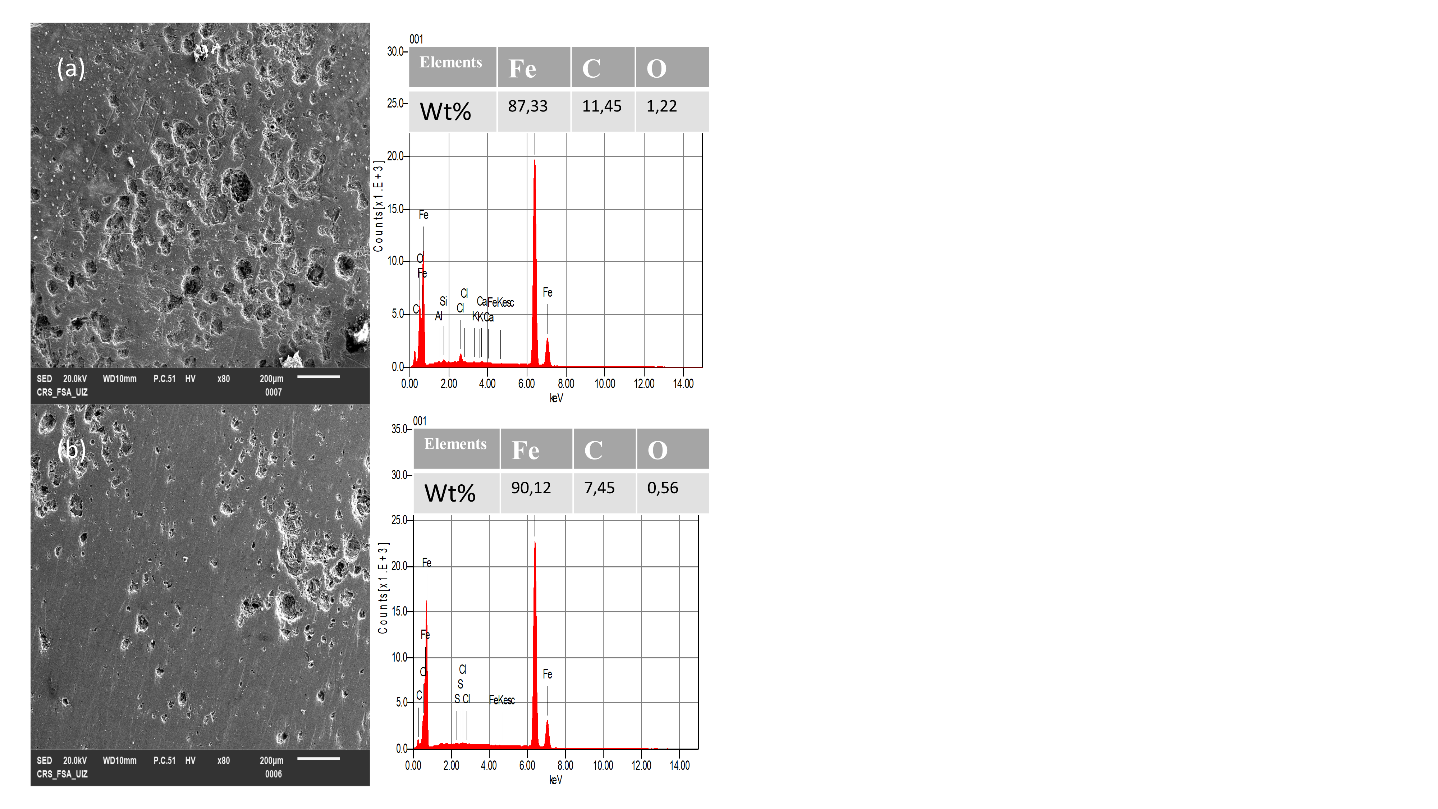
**

**Figure S9. SEM and EDS spectrum images of carbon steel: (a) immersion in 1 M HCl (b) immersion in 10^-3^M BM-01**

**
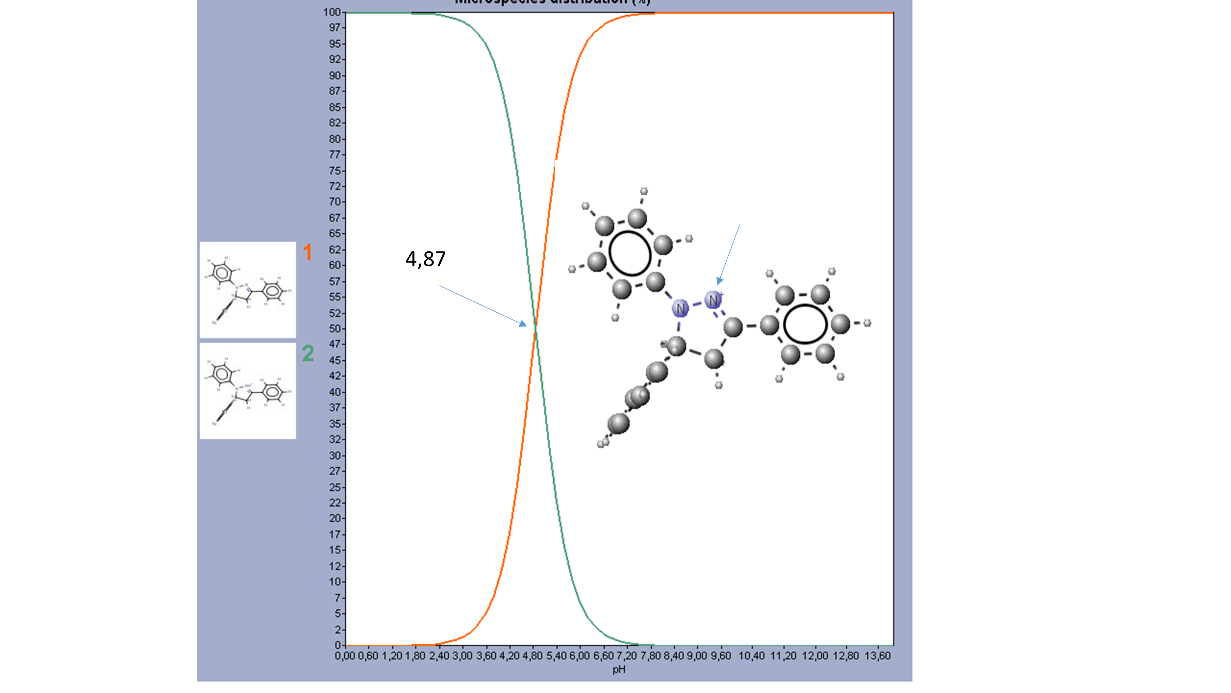
**

**Figure S10.** Sites favorable to protonation in an acid medium

**
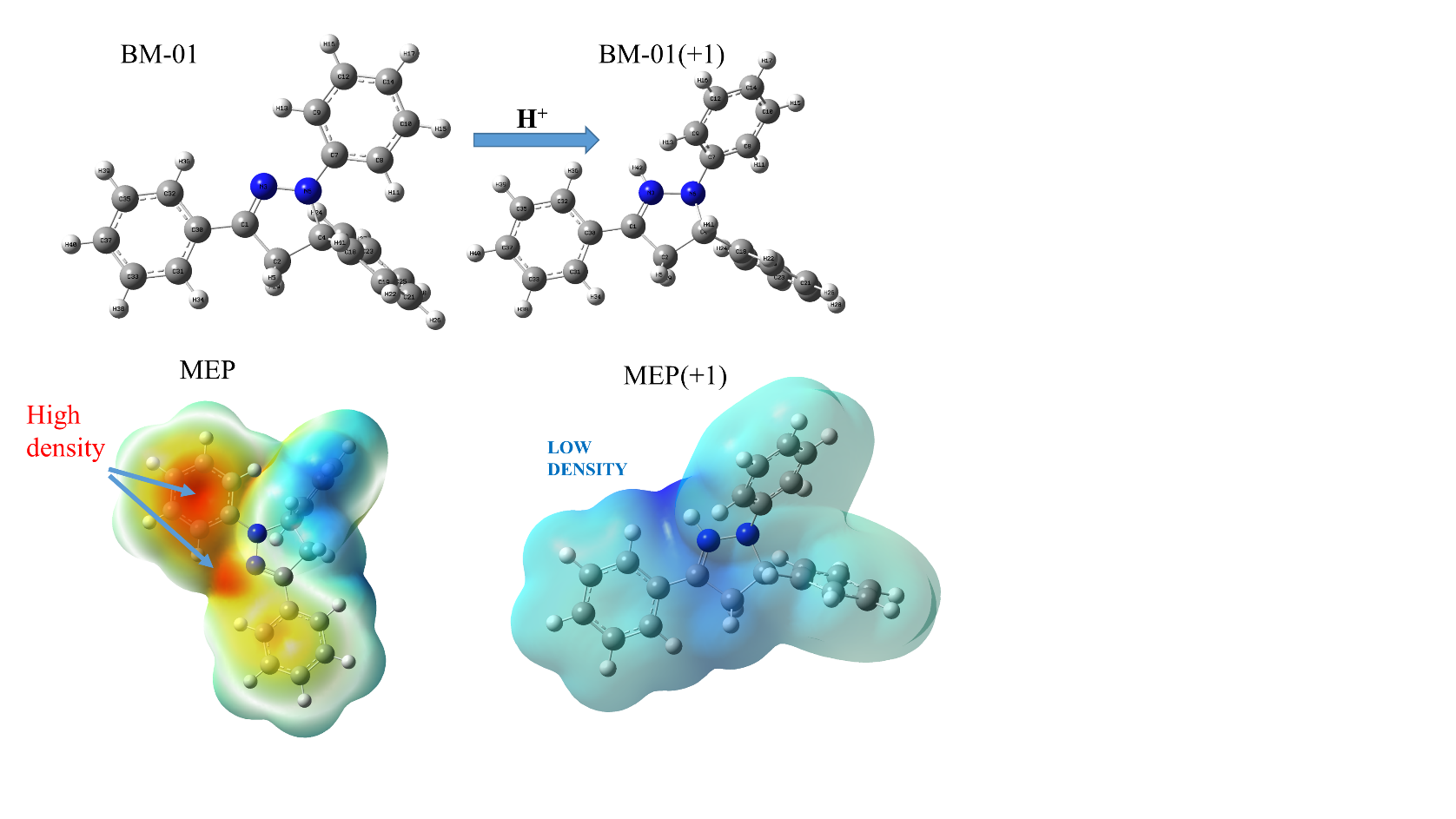
**

**Figure S11.** Optimized structures and distribution of neutral (BM-01) and protonated (BM-01 (+1)) PEMs

**Table S1**. Fukui functions, dual Fukui functions for BM-01

| Atoms | q(N) | q(N+1) | q(N-1) | f^+^ | f^-^ | f^2^ |
| --- | --- | --- | --- | --- | --- | --- |
| C1 | 0.22779 | 0.1243 | 0.34594 | -0.10349 | -0.11815 | 0.01466 |
| C2 | -0.43082 | -0.4197 | -0.44048 | 0.01112 | 0.00966 | 0.00146 |
| N3 | -0.27214 | -0.42151 | -0.25893 | -0.14937 | -0.01321 | -0.13616 |
| C4 | -0.02963 | -0.03161 | -0.0369 | -0.00198 | 0.00727 | -0.00925 |
| H5 | 0.23037 | 0.2038 | 0.26003 | -0.02657 | -0.02966 | 0.00309 |
| N6 | -0.25143 | -0.26309 | -0.06826 | -0.01166 | -0.18317 | 0.17151 |
| C7 | 0.1712 | 0.16634 | 0.16291 | -0.00486 | 0.00829 | -0.01315 |
| C8 | -0.26304 | -0.28055 | -0.17687 | -0.01751 | -0.08617 | 0.06866 |
| C9 | -0.25036 | -0.28778 | -0.1653 | -0.03742 | -0.08506 | 0.04764 |
| C10 | -0.18715 | -0.20207 | -0.18123 | -0.01492 | -0.00592 | -0.009 |
| C12 | -0.18846 | -0.19292 | -0.18274 | -0.00446 | -0.00572 | 0.00126 |
| C14 | -0.2503 | -0.29235 | -0.13034 | -0.04205 | -0.11996 | 0.07791 |
| C18 | -0.04312 | -0.0336 | -0.06057 | 0.00952 | 0.01745 | -0.00793 |
| C19 | -0.20767 | -0.21566 | -0.19799 | -0.00799 | -0.00968 | 0.00169 |
| C20 | -0.20968 | -0.21217 | -0.20691 | -0.00249 | -0.00277 | 0.00028 |
| C21 | -0.1945 | -0.19781 | -0.1881 | -0.00331 | -0.0064 | 0.00309 |
| C23 | -0.19275 | -0.19764 | -0.18586 | -0.00489 | -0.00689 | 0.002 |
| C25 | -0.20703 | -0.21758 | -0.1925 | -0.01055 | -0.01453 | 0.00398 |
| C30 | -0.09402 | -0.15004 | -0.11402 | -0.05602 | 0.02 | -0.07602 |
| C31 | -0.19406 | -0.25548 | -0.14665 | -0.06142 | -0.04741 | -0.01401 |
| C32 | -0.17835 | -0.26586 | -0.13691 | -0.08751 | -0.04144 | -0.04607 |
| C37 | -0.20273 | -0.33563 | -0.14247 | -01329 | -0.06026 | -0.07264 |

**
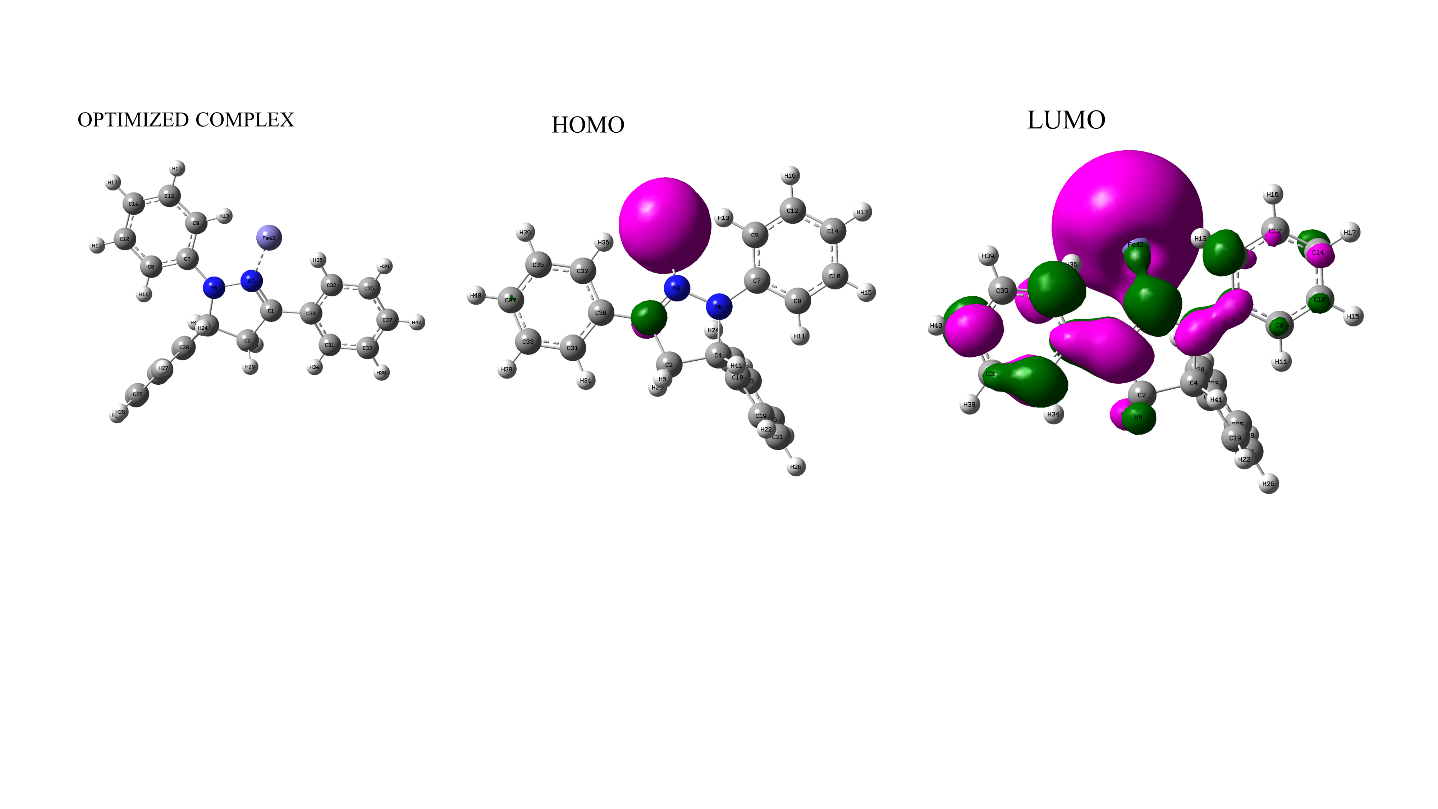
**

**Figure S12.** FMO distributions of the Fe-BM-01 complex

** Figure S13.** FDRs of the Fe (1 1 0) surface relative to the N3 atom of the neutral BM-01 at 298 and 328 K
